# Development and validation of a mobile application prototype for postoperative cardiac surgery

**DOI:** 10.1590/0034-7167-2023-0491

**Published:** 2024-10-07

**Authors:** Gabriele Cardoso Gonçalves Alves, Fabiola Leticia Damascena Amador, Vagner Rogério dos Santos, Rita Simone Lopes Moreira

**Affiliations:** I Universidade Federal de São Paulo. São Paulo, São Paulo, Brazil

**Keywords:** Mobile Applications, Cardiovascular Diseases, Postoperative Care, Self Care, Validation Study, Aplicaciones Móviles, Enfermedades Cardiovasculares, Cuidados Posoperatorios, Autocuidado, Estudio de Validación

## Abstract

**Objectives:**

to develop and validate the content, appearance, and semantics of a prototype application for monitoring patients in the postoperative period of cardiac surgery.

**Methods:**

this is a technological development study based on Contextualized Instructional Design. The content and appearance evaluation was conducted by a committee of specialists, and semantic validation was carried out by patients from a cardiac surgery outpatient clinic.

**Results:**

the application prototype consisted of 43 screens, validated by 17 health specialists, with content validity ratio and appearance validity index results of 0.86 and 0.99, respectively. For semantic validation, 10 patients participated in data collection, with a total content validity index of 0.978.

**Conclusions:**

the prototype of the “VivaCor PósOp” application demonstrated evidence of content, appearance, and semantic validity, with the potential to stimulate self-care in patients in the postoperative period of cardiac surgery.

## RESUMEN

**Objetivos:**

desarrollar y validar los contenidos, la apariencia y la semântica del prototipo de una aplicación para el seguimiento de pacientes en el postoperatorio de cirugía cardíaca.

**Métodos:**

estudio de desarrollo tecnológico basado en el Diseno Instruccional Contextualizado. La evaluación de contenido y apariencia fue realizada por un comité de especialistas, y la validación semântica fue realizada por pacientes del ambulatorio de cirugía cardíaca.

**Resultados:**

el prototipo de la aplicación resultó en 43 pantallas, validadas por 17 especialistas en salud, con resultados de razón de validez de contenido e índice de validez de apariencia totales de 0,86 y 0,99, respectivamente. En cuanto a la validación semântica, 10 pacientes participaron en la recolección de datos, con un índice de validez de contenido total de 0,978.

**Conclusiones:**

el prototipo de la aplicación “VivaCor PósOp” presentó evidencias de validez de contenido, apariencia y semântica, con potencial para estimular el autocuidado en pacientes en el postoperatorio de cirugía cardíaca.

## INTRODUCTION

With the advancement of technologies and the aging of the global population, the use of digital health strategies has become an increasingly crucial necessity in the global context^(1)^. Thus, the growing prevalence of non-communicable chronic diseases (NCDs), combined with the search for resources that promote self-care and use technology as a tool in the health sector, has driven and strengthened actions in the field of digital health^(1)^.

To improve the provision of health care and overcome some of the barriers in global health systems, the use of digital technologies, such as eHealth, which relies on the use of information and communication technologies (ICT) applied to health, and mHealth, which focuses on the use of ICT supported by mobile devices, has emerged as promising strategies^(2, 3, 4)^.

In this scenario, the integration of ICTs in the health sector has become essential to address the challenge of NCDs and multimorbidities^(1)^. Among the NCDs, cardiovascular diseases (CVDs) are the leading cause of death worldwide, accounting for approximately 17.6 million deaths in 2016^(5)^. Regarding surgical procedures for CVDs, the Brazilian Society of Cardiology (SBC) reported a 64% increase between 2008 and 2019, which represents a significant impact both in terms of costs and on the health status of the individual^(6)^.

Considering the surgical periods, the postoperative period is a complex and extremely delicate time, especially in major surgeries, where incidents such as pain, thrombotic events, fatigue, and prolonged convalescence are frequently observed^(7)^. Therefore, to assist patient recovery, cardiac rehabilitation (CR) programs have been increasingly employed^(8)^. CR is an intervention based on the actions of a multidisciplinary team, aiming to reduce the physiological and psychological impacts of CVDs, as well as assist in the clinical stabilization of the patient^(8)^.

In this sense, CR programs have key elements based on patient self-care and self-management strategies^(8)^. Focusing on the self-care of patients with CVDs, the use of mobile applications has been a growing trend worldwide^(3)^. Thus, the use of eHealth and mHealth strategies is considered an innovative alternative to traditional health education methods for delivering information in an accessible manner^(9, 10)^. Given this, the use of mobile applications to structure patient-centered care proves to be a relevant approach, providing essential information about the disease during the rehabilitation process^(3)^.

However, even with this context, a search for applications in the Google® Play Store and Apple® Store did not find resources aimed at patients in the postoperative period of cardiac surgery in the Portuguese language. Therefore, considering the progressive increase in internet use, especially in age groups over 45 years^(2)^, the decision was made to develop an application to monitor patients in the postoperative period of cardiac surgery, with the aim of providing a tool capable of storing the patient’s clinical history, consequently reducing misinformation, recurrent readmissions, and having a lower impact on health costs.

## OBJECTIVES

To develop and validate the content, appearance, and semantics of a prototype application for monitoring patients in the postoperative period of cardiac surgery.

## METHODS

### Ethical Aspects

The research protocol, originating from a master’s thesis, was reviewed and approved by the Human Research Ethics Committee of a federal university in São Paulo.

### Study Design, Period, and Location

This is a technological development and validation study based on Contextualized Instructional Design^(11)^, aimed at creating and analyzing evidence of content, appearance, and semantic validity of a mobile application prototype. The study was conducted in a virtual environment with expert jurors between October 2022 and March 2023, and in the cardiac surgery outpatient clinic of a federal university in São Paulo, between March and April 2023, with patients. The data analyzed in this study are derived from a master’s thesis, guided by the Guideline for Reporting for Intervention Development Studies (GUIDED)^(12)^.

### Population, Inclusion, and Exclusion Criteria

For the content and appearance validation stage of the application prototype, 200 health professionals (nurses, physiotherapists, nutritionists, pharmacists, and doctors) were invited, with 40 from each area. Recruitment was preceded by profile characterization, evaluating the area of expertise, years of professional experience, and academic qualifications, considering a minimum score of five based on the adapted Fehring’s Expert Validation Model Classification System^(13, 14)^.

Selected professionals received an invitation letter via email, sent through the RedCap® platform, with all study information, including its objectives, target audience, questionnaires to be answered, and affiliated institution, along with the Informed Consent Form (ICF). Once the invitation was accepted, the jurors received a second email containing the questionnaires and a link to access Google Drive®, where they found the video mockup (.mp4) and a Portable Document Format (PDF) version with the application screens.

After recruitment, jurors who did not meet the minimum score of 5 in the Fehring’s Expert Validation Model Classification System^(13, 14)^, and those who did not complete any of the questionnaires within 4 months, despite receiving up to 3 reminders, were excluded. Additionally, those who did not justify their evaluations and those who expressed significant doubts regarding the population or technology evaluated in their comments, compromising their judgment, were excluded.

For the semantic validation phase, patients who were in the immediate or late postoperative period of cardiac surgery performed at the reference hospital of a federal university in São Paulo, who were being followed up at the outpatient clinic of the same institution, over 18 years of age, literate, and who agreed to participate in the study by signing the ICF, were included. Patients with visual impairments or any limitations that could compromise the evaluation of the screens were excluded from the study.

### Study Protocol

**Figure 1:**
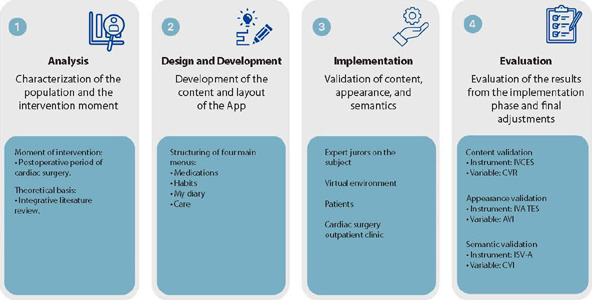
Study Stages According to the Contextualized Instructional Design Methodology

The methodology adopted for technological development was based on the concept of Contextualized Instructional Design (CID)^(11)^. The methodological process consisted of four interlinked phases executed cyclically^(11)^, comprising the following stages: analysis, design and development, implementation, and evaluation, as shown in Figure 1.

### Phase 1: Analysis

The application prototype was developed within the context of a research group composed of health professionals from a multidisciplinary team and technology professionals. During the analysis stage, the researchers characterized the population, opting for an application aimed at patients in the immediate or late postoperative period of cardiac surgery. They also considered it important to update the institution’s discharge manual data after cardiac surgery.

### Phase 2: Design and Development

The content of the application prototype was developed based on an integrative literature review conducted by the authors in 2022 for this purpose. Articles, guidelines, and recommendations related to CR, the prevention of CVDs, and the postoperative period of cardiac surgery published in the last 10 years were included. From this search, the main areas of focus were identified, which constituted the following icons of the application prototype: medications, habits, my diary, and care. Subsequently, the application prototype was structured using the Canva® platform. The initial model, developed by the author in partnership with a second reviewer, was presented during research group meetings. After two presentations, the final model was reached, consisting of 43 screens.

### Phase 3: Implementation

The implementation phase involved the evaluation of content and appearance by a committee of specialists^(15)^ and semantic validation by a pilot group of patients. Validation consists of verifying the presence of validity attributes, meaning that through this process, the technology is considered adequate to fulfill its proposed task^(15)^.

Since there was no specific statistical test to evaluate content validity, a qualitative approach was adopted, with a committee of specialists^(16)^ composed of six to twenty jurors, according to previous studies^(17)^. The number of professionals on the committee was determined based on Lawshe’s content validity ratio (CVR), which measures the specialists’ agreement regarding the essentiality of items^(18)^.

Content validation was conducted using the Instrument for Validation of Educational Content in Health (Portuguese acronym: IVCES)^(19)^. The responses in the IVCES were categorized according to Lawshe’s proposed categories: “not necessary”, “useful but not essential”, and “essential”^(18)^. The CVR was calculated for each item and domain separately.

Appearance validation was conducted using the Instrument for Validation of Appearance of Educational Technology in Health (Portuguese acronym: IVATES)^(20)^. The appearance validity index (AVI) was calculated for each of the twelve items in the IVATES and for the questionnaire as a whole. The minimum acceptable concordance value was 0.78 for an item to not require revision^(20)^.

Semantic validation plays a crucial role in the evaluation of educational technologies, allowing for adjustments in layout and content, aligning with the CID methodology^(11)^. This analysis involves the target audience, with special attention to less favored social strata, through a pre-test, ensuring that the items are understandable and suitable for the educational technology’s target population^(21, 22)^. Data collection involved two questionnaires: the first addressed sociodemographic and identification aspects, while the second, adapted from Oliveira’s study^(23)^, consisted of five axes structured based on the Suitability Assessment of Materials (SAM)^(24)^. The Instrument for Semantic Validation (ISV), an adapted questionnaire, uses a four-point Likert scale to evaluate items. The reliability of the adapted ISV was assessed using McDonald’s omega coefficient (w), with values of w > 0.70 indicating the reliability of the set of factors^(25)^.

To evaluate the results of the semantic validation questionnaire, the Content Validity Index (CVI)^(17)^ was used, which reflects the proportion of evaluators in agreement with the questionnaire items, calculated individually for each item, for the thematic axes, and for the instrument as a whole.

### Phase 4: Evaluation

The evaluation phase involved analyzing the results obtained during the implementation phase. Data collection was conducted via the RedCap® platform for both jurors and patients, and the data were stored on the server and later retrieved in Microsoft Excel® spreadsheets. For validation, a simple statistical analysis was performed, calculating the CVR, AVI, CVI, and McDonald’s ω from the scores of the IVCES, IVATES, and adapted ISV questionnaires. For this data analysis, the theoretical basis described by Field^(25)^ was used, with a statistical significance value set at 5% (p ≤ 0.05), using SPSS Statistics software, version 28.0 (IBM Corp., Armonk, NY, USA).

## RESULTS

**Figure 2:**
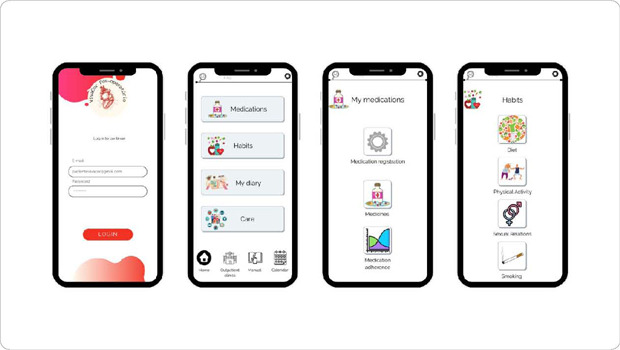
Screens of the VivaCor Pós-op Application Prototype

An integrative literature review identified four guidelines: one from the SBC^(26)^, one from the American Heart Association (AHA)^(27)^, and two from the European Society of Cardiology (ESC)^(28, 29)^, focused on CR, prevention of CVDs, and postoperative care for cardiac diseases. Additionally, four review articles on CR^(8, 30, 31, 32)^ and a recommendation from the AHA^(33)^ were included. Based on these findings, the prototype icons were constructed, covering the following topics: wound care, medications, physical activity, sexual relations, diet, smoking and alcohol use, blood pressure control, weight, and warning signs for seeking immediate medical attention. Figure 2 represents the main screens of the application prototype.

For the validation phase by specialists, of the 200 jurors invited, 36 agreed to participate in the study, and of these, only 27 completed all the proposed questionnaires. To ensure the quality of the sample, responses from professionals who scored 5 or less in Fehring’s Expert Validation Model Classification System^(13)^ (n = 6) were excluded, as well as those from jurors who made it clear in their comments that they did not understand the evaluation proposal, target audience, or the questionnaire (n = 4).

The sample consisted of 17 jurors, the majority of whom were nurses (n = 10), followed by physiotherapists (n = 4), a nutritionist (n = 1), and a pharmacist (n = 1). All participants had expertise in cardiology, with 6 holding doctoral degrees, 6 holding master’s degrees, 4 holding postdoctoral degrees, and 1 being a specialist. The scores according to Fehring’s criteria ranged from 6 to 13 points, with an average of 10.7 points. Regarding the age of the specialists, the average was 36 years, the median was 40.4 years, and the minimum and maximum values were 28 and 63 years, respectively. This detailed characterization can be found in the Supplementary Material.

Considering the relationship between CVR and the number of evaluating specialists established by Lawshe, to ensure that the agreement is unlikely due to chance, it was determined that the minimum CVR value was 0.49^(18)^. The CVR results by axis were: Objectives = 0.86; Structure = 0.82; Relevance = 1. Regarding the evaluation of the CVR by item, a variation between 0.53 and 1 was observed. The detailed scores can be found in the Supplementary Material.

The jurors were invited to provide comments on questions where there was partial or total disagreement, in addition to having the opportunity to make general comments at the end of the questionnaire. All pertinent comments were considered and discussed in a research group, and the screens were revised by the author after the modifications. Subsequently, two independent jurors evaluated the altered topics and checked for possible typographical errors. In addition to content validation, the specialists also validated the appearance of the prototype interface using the IVATES, calculating the IVA, which ranged from 0.94 to 1 (Table 1). Similarly, the jurors were invited to comment on the alternatives for which they reported “partially disagree”, “disagree”, or “totally disagree”. Thus, for this questionnaire, we received only two suggestions: (i) the separation of the “mood” and “pain” sections; (ii) the increase in the size of the figures in the “sexual activity” section. Both suggestions were accepted, and the layout was modified accordingly.

**Table 1:** Appearance Validity Index of the application screens using Validation of Appearance of Educational Technology in Health, São Paulo, São Paulo, Brazil, 2023

IVATES Items	AVI-I	AVI-T
1. The illustrations are appropriate for the target audience.	1	
2. The illustrations are clear and convey ease of understanding.	0.941	
3. The illustrations are relevant for the target audience’s understanding of the content.	1	
4. The colors of the illustrations are suitable for the type of material.	1	
5. The shapes of the illustrations are suitable for the type of material.	1	
6. The illustrations depict the daily life of the target audience of the intervention.	1	
7. The arrangement of the figures is in harmony with the text.	1	
8. The figures used elucidate the content of the educational material.	1	
9. The illustrations help in presenting the theme and follow a logical sequence.	1	
10. The illustrations are in adequate quantity in the educational material.	1	
11. The illustrations are of appropriate size in the educational material.	0.941	
12. The illustrations help in changing the behaviors and attitudes of the target audience.	1	0.990

*IVATES - Validation of Appearance of Educational Technology in Health; AVI-I – Item appearance validity index; AVI-T – Total Appearance Validity Index.*

The study involved 12 eligible patients at a cardiac surgery outpatient clinic, collecting data on two separate occasions, with the participation of 10 patients. The majority of participants were male (90%), with 40% having incomplete primary education and an average age of 66.07 years. There was a high prevalence of self-reported comorbidities, notably systemic arterial hypertension (90%), dyslipidemia (70%), and heart failure (40%). Regarding cardiac surgery, 30% of the patients had undergone the procedure 1-2 years ago, 20% had it 3-4 years ago, and the remaining had it more than 5 years ago. Detailed patient characterization is available in the Supplementary Material.

Prior to semantic validation, the internal consistency of the instrument’s domains was analyzed using McDonald’s omega coefficient, as shown in Table 2. In terms of semantic validation, no item was considered inadequate, but three questions were classified as partially adequate. The CVI results showed a high overall score (0.978). Detailed evaluations can be found in the Supplementary Material.

**Table 2:** Content Validity Index and McDonald’s Omega of the Adapted Semantic Validation Instrument, São Paulo, São Paulo, Brazil, 2023

Domains of the Semantic Validation Instrument	CVI	ω
Objectives	0.967	0.971
Organization	0.980	0.837
Language	1	0.892
Appearance	1	0.821
Motivação	0.933	0.687

*CVI – Content Validity Index; ω – McDonald’s omega coefficient.*

## DISCUSSION

The present study addressed the validation of the content, appearance, and semantics of the “VivaCor PósOp” prototype, presenting positive results that support its application as an educational health intervention.

Despite the vast potential to be explored in the sphere of mHealth, technical challenges can affect the acceptability and viability of telehealth systems, such as the need for high-quality internet access^(34)^. In the Brazilian context, internet access has grown in recent years, reaching about 59 million households (82%) in 2021. However, it is important to note that the distribution of fixed internet access in the country is not uniform, being predominant in the South and Southeast regions and among families in classes A and B^(35)^.

Thus, it is essential that telehealth interventions be adapted to the needs and characteristics of the target audience, considering the different realities existing in the country, especially in Brazil, which presents a wide diversity of socioeconomic and geographical realities.

For this validation, a multidisciplinary team was invited, with the majority participation of nurses, followed by representatives from other areas. However, there was no acceptance of collaboration from medical professionals. Nevertheless, it is important to highlight that other studies that addressed the validation of health applications presented promising results even without the evaluation of medical professionals^(36, 37)^, reinforcing the strength of multidisciplinary knowledge in health care. Furthermore, content validation by specialists can be considered a fundamental resource to support the design of telehealth interventions^(38)^. Therefore, in light of the multidisciplinary care of patients in the postoperative period of cardiac surgery, it is suggested that ongoing studies involve other experts, such as doctors and technology specialists. This approach allows for a comprehensive understanding of the needs and possibilities for developing technological health products.

In line with the methodology adopted for the validation of “VivaCor PósOp”, the study conducted by Fernández-Gutiérrez et al.^(38)^ also focused on the validation of the mICardi application, designed for the self-care of patients with heart failure, aiming to improve quality of life (QoL). In this validation, the CVI was calculated for each screen of the application, resulting in a total CVI of 0.851, indicating its effectiveness in improving patients’ QoL and promoting health literacy. Similar to the cited study, the “VivaCor PósOp” prototype was developed with methodological rigor, involving the participation of specialists in the field, which strengthens its credibility. It is crucial that these resources are constructed and validated using robust methodologies to ensure their reliability and transparency^(39)^.

Although there are technical challenges, telerehabilitation was considered by users to be an alternative and promising model to center-based CR, as shown in a systematic review by Subedi et al., which observed high acceptance of telerehabilitation by patients due to its flexibility and ease of access. Additionally, interventions that offered self-monitoring sessions and individualized feedback were positively evaluated by patients^(34)^. Therefore, future studies using “VivaCor PósOp” with patients undergoing cardiac surgery are necessary to assess acceptance, self-monitoring, and health follow-up.

In this context, the low motivation of patients is a common issue in CR. The self-determination theory highlights the importance of satisfying psychological needs related to exercise, which is associated with greater self-determined motivation and more effective exercise behavior^(40)^. Concurrently, promoting health literacy represents a significant opportunity to transform health education, requiring a proactive approach from patients^(41)^. In this sense, it is important to highlight the collaboration between health professionals and technology, as well as the active participation of patients in the development of eHealth resources^(42)^. This user-centered approach allows for a better understanding of patients’ needs, as occurred in the second phase of the validation of the “VivaCor PósOp” prototype, which collected opinions from future users through a structured questionnaire.

The semantic validation of the “VivaCor PósOp” prototype involved the participation of 10 patients. In the initial analysis, the internal consistency of the five domains of the semantic validation instrument was evaluated using McDonald’s omega coefficient^(25)^. The domains of objective, organization, language, and appearance presented satisfactory omega values, all above 0.70, as indicated by Campo-Arias et al.^(43)^. However, in relation to the motivation domain, a McDonald’s omega coefficient of 0.687 was observed, indicating a relatively low correlation between the items in this domain. Additionally, patients gave the lowest score to this domain, with a CVI score of 0.933. It is important to note that the construct of motivation is multifaceted, encompassing the individual’s disposition regarding their behavior and its consequences^(44)^.

Thus, a relevant point to consider is the time elapsed since the surgeries of the sample patients, with about 50% having undergone surgical intervention more than five years ago. Motivation is a dynamic characteristic, sensitive to variations in personal and social circumstances^(44)^, suggesting that the prolonged postoperative period may have influenced the results observed in this domain. Therefore, it is crucial to implement various strategies to promote motivation.

A systematic review conducted by Chang et al. explored health interventions based on the Information-Motivation-Behavioral Skills (IMB) Model, recognizing these components as determinants for adopting healthy behaviors and behavioral change. After analyzing twelve studies, Chang et al. found that the most common strategies were the use of informational pamphlets, motivational interviews, and educational videos. These approaches showed positive results, with an improvement in behavior change observed in 83.33% of the studies analyzed^(44)^.

In this sense, the combination of remote monitoring and individual follow-up has shown positive results in reducing patient mortality and hospitalization, especially in the short term^(45)^. Additionally, robust technological strategies combined with multidisciplinary approaches can contribute to promoting behavioral change and improving health outcomes. Therefore, it is recommended to include models with personalized counseling, specific goal setting, measures to promote psychosocial well-being, and comprehensive, up-to-date guidance^(39, 46)^. Ensuring the reliability and transparency of data in mobile applications is of fundamental importance^(39)^.

Finally, in the patient journey, the transition of care from the hospital to the home environment plays a crucial role in the individual’s recovery after cardiac surgery. In this context, the implementation of an mHealth tool specifically designed to meet the needs of this population emerges as an innovative solution to assist this process. It is from this perspective that the “VivaCor PósOp” application prototype was developed, showing evidence of content and appearance validity.

### Study limitations

The study’s limitations include the unequal distribution of expertise among health professionals, with a predominance of nurses, a small number of patients in the pre-test, and the evaluation of screens without direct interaction or usability testing. The next phase of the study will focus on the application’s programming, addressing details related to systems, browsers, and screen adaptation to the resources used. This will enable future investigations to obtain usability data as well as the clinical validation of the “VivaCor PósOp” application with patients.

### Contributions to the Field

The development of mHealth resources adapted to the specific needs and characteristics of the target audience, considering the various socioeconomic and geographic realities in the Brazilian context, emerges as a fundamental strategy. These resources have the potential to facilitate the transition from hospital to home care, providing support to both the patient and the family through information validated by specialists in the field, promoting a more personalized and effective health experience.

## CONCLUSIONS

The study focused on developing the “VivaCor PósOp” application prototype, highlighting its ability to promote self-care in patients after cardiac surgery. Addressing crucial elements of the postoperative period and backed by scientific evidence, the application obtained satisfactory validations in both content (CVR=0.86) and appearance (AVI=0.99). The semantic validation, conducted with post-cardiac surgery patients, reinforced its reliability, presenting a total CVI of 0.978. These results underscore the importance of using validated educational technologies like “VivaCor PósOp” in the health scenario, emphasizing its relevance as a promising tool to promote self-care and ensure effective follow-up during postoperative recovery.

## Supplementary Material

0034-7167-reben-77-05-e20230491-suppl01

## Data Availability

https://doi.org/10.48331/scielodata.ILIRZR
