# Supplementary material for: Development and validation of a mobile application prototype for postoperative cardiac surgery
Source: Rev Bras Enferm. 2024 Oct 7;77(5):e20230491. doi: 10.1590/0034-7167-2023-0491 (PMC11458145; doi:10.1590/0034-7167-2023-0491)
Supplement: 0034-7167-reben-77-05-e20230491-suppl01 [file 0034-7167-reben-77-05-e20230491-suppl01.pdf]

Quadro 1 - Critérios adaptados do Sistema de Classificação de especialistas do Modelo de Validação Fehring, São Paulo, São Paulo, Brasil, 2024

Critérios Adaptados de Fehring	Pontuação
Mestrado em ciências da saúde ou áreas correlatas	4
Mestrado em ciências da saúde com foco em cardiologia, tecnologia ou educação em saúde	1
Publicação de artigo nas áreas de cardiologia, tecnologia ou educação em saúde em periódicos de referência	2
Artigo publicado sobre cardiologia, tecnologia ou educação em saúde com conteúdo relevante para a área	2
Doutorado em ciência da saúde	2
Experiência clínica, pelo menos um ano em cardiologia e áreas correlatas	2
Certificado (especialização) relevante prática no campo de cardiologia	2

Tabela 1 - Caracterização dos jurados especialistas, São Paulo, São Paulo, Brasil, 2024

Variáveis		Frequência (%)
**Sexo**	Feminino	76,5
	Masculino	23,5
**Idade**	20 - 30	17,65
	30 - 40	41,18
	40 - 50	23,53
	50 - 60	11,76
	60 - 70	5,88
**Maior grau acadêmico**	Especialização	5,88
	Mestrado	35,29
	Doutorado	35,29
	Pós-doutorado	23,53
**Área de atuação**	Cardiologia	100
	Educação em saúde	29,41
	Tecnologia	11,76
	Outro	17,65
**Tempo de atuação profissional**	4 – 5 anos	5,88
	> 5 anos	94,22
**Pontuação segundo critérios de Fehring**	6	11,76
	8	11,76
	10	11,76
	11	17,65
	12	17,65
	13	29,41

Quadro 1 - Comentários e sugestões dos especialistas, São Paulo, São Paulo, Brasil, 2024

Eixo	Comentário	Ação/ Justificativa
Objetivos	"Seria importante fornecer mais detalhes a respeito da atividade física, com orientações que vão além da caminhada. Abordar a questão de percepção de esforço para monitoramento da intensidade do exercício"	Reformulação do feedback ao paciente, desencorajando a atividade física segundo classificação do esforço.<br><br>Inicialmente optou-se por seguir apenas com orientações de atividade leves, considerando a autogestão do paciente
	"Utilizar outros meios de difusão dos conteúdos como áudio ou vídeo, por exemplo"	Integração do VivaCor Cast, um Podcast sobre doenças cardiovasculares ao aplicativo<br>Criação de vídeos com os conteúdos do App
	"Fornecer ao paciente, feedbacks mais personalizados, para os parâmetros de monitoramento, isso pode auxiliar na compreensão e reflexão sobre a importância das ações"	Já estavam estipulados feedbacks para todas as variáveis de autogerenciamento (peso, pressão arterial, humor e dor)
Estrutura	"Adequar a linguagem, focando no uso dos seguintes termos: como ingerir, comprovado cientificamente, impactado, planos e outros"	Substituição dos termos e revisão da linguagem utilizada
	"Separar a tabela de dor e humor, pode gerar confusão"	Separação das seções dor e humor
	"Adicionar questionário validado para avaliação do humor, a escala de faces consiste em uma forma de avaliação incompleta e altamente subjetiva"	Adicionada a escala de Brums para avaliação de humor dos usuários.
	"Seria importante que não estimulassem os pacientes a identificar os medicamentos de uso contínuo pela cor do comprimido, visto que essa característica pode variar conforme cada laboratório"	Compreensão inadequada da função. As cores são relativas às *Tags* coladas na caixa de medicação durante as consultas de enfermagem ambulatoriais, não aos medicamentos.<br>Alteração da configuração da tela, para que essa função fique explícita.

	“No ícone ‘Sinais de Alarme’ sugiro modificar o item 4 ‘aumento de tosse e inchaço’, escrevendo-os de forma separada, como por exemplo ‘aumento de tosse e falta de ar’ e ‘aumento do inchaço nas pernas ou barriga’ ”	Alterado o item 4 da seção ‘Sinais de alarme’

Tabela 2 - Razão de Validade de Conteúdo das telas do aplicativo por meio do IVCES, São Paulo, São Paulo, Brasil, 2024

Item do IVCES	RVC-I	RVC-E
**Objetivos**		
1.Contempla o tema proposto	0,88	0,86
2. Adequado ao processo ensino-aprendizagem	1,00	
3.Esclarece dúvidas sobre o tema abordado	0,88	
4.Proporciona reflexão sobre o tema	0,88	
5. Incentiva mudança de comportamento	0,65	
**Estrutura**		
6.Linguagem adequada ao público-alvo	0,76	0,82
7.Linguagem apropriada ao material educativo	1,00	
8.Linguagem interativa, permitindo envolvimento ativo no processo educativo	0,53	
9. Informações corretas	0,88	
10. Informações objetivas	0,88	
11. Informações esclarecedoras	1,00	
12. Informações necessárias	0,76	
13. Sequência lógica das ideias	0,88	
14. Tema atual	1,00	
15. Tamanho do texto adequado	0,53	
**Relevância**		
16. Estimula o aprendizado	1,00	1
17. Contribui para o conhecimento na área	1,00	
18. Desperta interesse pelo tema	1,00	

Tabela 3 - Caracterização dos pacientes, São Paulo, São Paulo, Brasil, 2024

Variáveis		N	Frequência (%)
**Sexo**	Feminino	1	10
	Masculino	9	90
**Idade**	55 - 60	1	10
	60 - 65	3	30
	65 - 70	5	50
	70 - 75	1	1
**Escolaridade**	EF incompleto	4	40
	EF completo	2	20
	EM incompleto	1	10
	EM completo	2	20
	ES incompleto	0	0
	ES completo	1	10
**Acesso à internet**	Sim	6	60
	Não	4	40
**Aparelho celular próprio**	Sim	4	40
	Não	6	60
**Comorbidades**	2	3	30
	3	6	60
	4	1	10
**Tabagismo**	Sim	0	0
	Não	10	100
	Ex-fumante	5	50
**Uso de bebidas alcoólicas**	Sim	1	10
	Não	9	90
**Cirurgia Cardíaca**	1 a 2 anos	3	30
	3 a 4 anos	2	20
	5 a 6 anos	3	30
	7 a 8 anos	1	10
	Mais de 10 anos	1	10

Tabela 4 - Índice de Validade de Conteúdo para questões e domínios do Instrumento de Validação Semântica Adaptado, São Paulo, São Paulo, Brasil, 2024

Item do IVS	IVC* item	IVC* total
**Objetivos**		
1. Atende aos objetivos de saúde dos pacientes em pós-operatório de cirurgia cardíaca	0,9	
2. Ajuda no cotidiano, após a cirurgia cardíaca e alta hospitalar	1	0,967
3. Proporciona conhecimento sobre o pós-operatório, reabilitação e cuidados com o coração	1	
**Organização**		
4. As telas do aplicativo são atraentes	1	

5. O tamanho do texto nos títulos e no conteúdo está adequado	1	0,980
6. Os tópicos têm sequência	1	
7. Os ícones do aplicativo retratam tópicos importantes	1	
8. O material dificulta em alguma parte a visualização dos tópicos	0,9	
**Linguagem**		
9. O vocabulário utilizado nas telas do aplicativo é acessível	1	
10. O texto está claro	1	
11. As frases são explicativas	1	1
**Aparência**		
12. As imagens são simples e atrativas	1	
13. As telas do aplicativo retratam a orientação de forma adequada	1	1
14. As imagens auxiliam na compreensão do texto	1	
15. As telas parecem organizadas e seguem uma lógica	1	
**Motivação**		
16. Na sua opinião, qualquer participante que utilizar o aplicativo vai entender do que se trata	1	
17. Você se sentiu motivado a utilizar o aplicativo	1	0,933
18. O aplicativo pode ser uma ferramenta importante após a alta hospitalar	0,8	

*IVC: Índice de validade de conteúdo

Tabela 5 - Caracterização da amostra em relação às respostas para cada questão do Instrumento de Validação Semântica, São Paulo, São Paulo, Brasil, 2024

**Questão**	**Resposta**							
	**Inadequado**		**Parcialmente adequado**		**Adequado**		**Totalmente adequado**	
	**n**	**%**	**n**	**%**	**n**	**%**	**n**	**%**
1	0	0,00	1	10,00	3	30,00	6	60,00
2	0	0,00	0	0,00	4	40,00	6	60,00
3	0	0,00	0	0,00	4	40,00	6	60,00
4	0	0,00	0	0,00	4	40,00	6	60,00
5	0	0,00	0	0,00	6	60,00	4	40,00
6	0	0,00	0	0,00	6	60,00	4	40,00
7	0	0,00	0	0,00	4	40,00	6	60,00
8	0	0,00	1	10,00	4	40,00	5	50,00

9	0	0,00	0	0,00	5	50,00	5	50,00
10	0	0,00	0	0,00	5	50,00	5	50,00
11	0	0,00	0	0,00	5	50,00	5	50,00
12	0	0,00	0	0,00	3	30,00	7	70,00
13	0	0,00	0	0,00	4	40,00	6	60,00
14	0	0,00	0	0,00	2	20,00	8	80,00
15	0	0,00	0	0,00	4	40,00	6	60,00
16	0	0,00	0	0,00	4	40,00	6	60,00
17	0	0,00	0	0,00	6	60,00	4	40,00
18	0	0,00	2	20,00	3	30,00	5	50,00

Quadro 3 - Comentarios dos pacientes, São Paulo, São Paulo, Brasil, 2024

**Domínio**	**Comentário**
Objetivos	Acho que ainda faltam pontos importantes como fornecer feedbacks personalizados ao paciente, providenciar encaminhamentos, ter contato direto com a equipe de saúde.
Organização	Pensar em outros formatos, como um website
Motivação	Talvez seja difícil usar no celular
	Vídeo e áudio podem ajudar

Figura 1 - Versão final das telas do protótipo do aplicativo VivaCor Pós-Op., São Paulo, São Paulo, Brasil, 2024

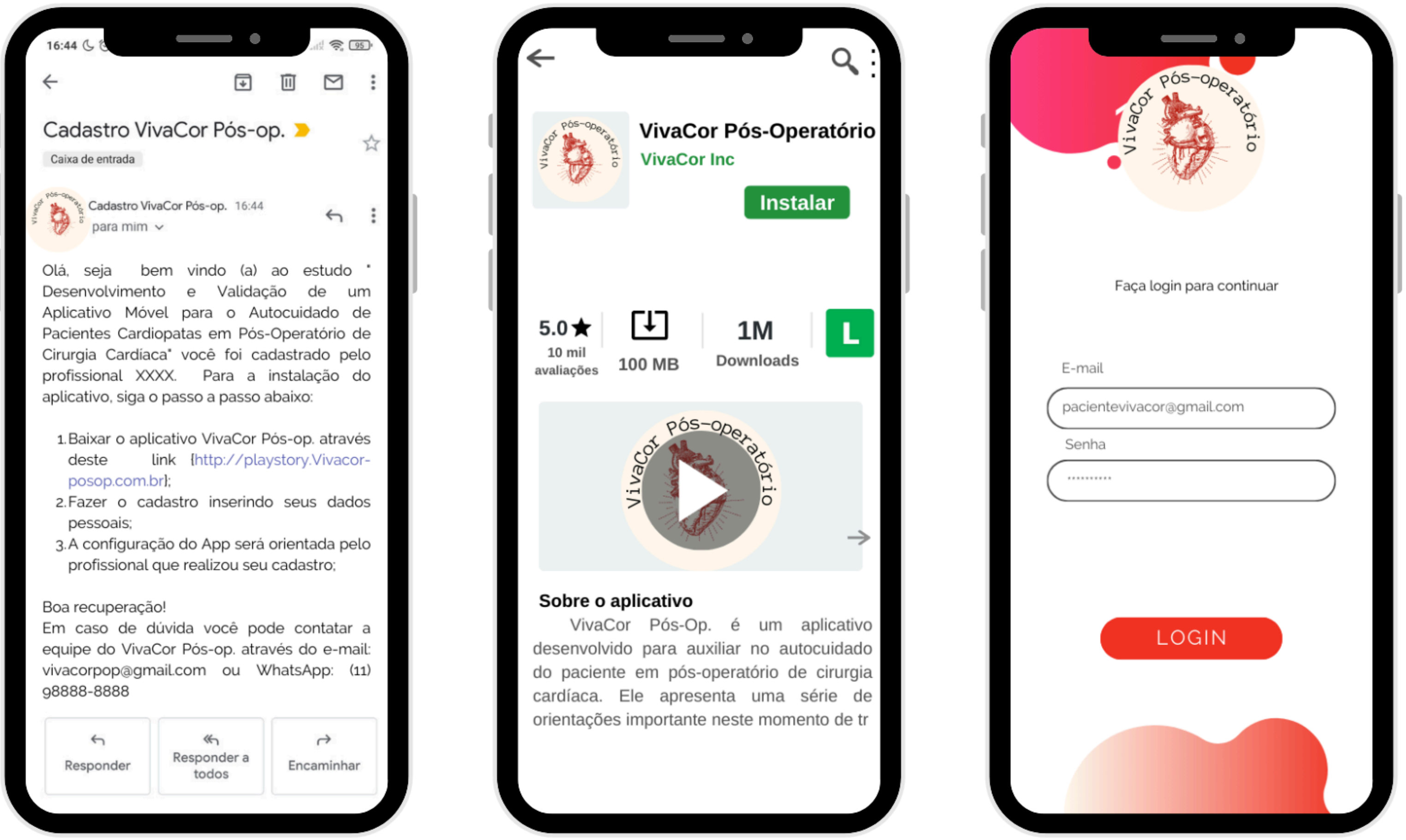

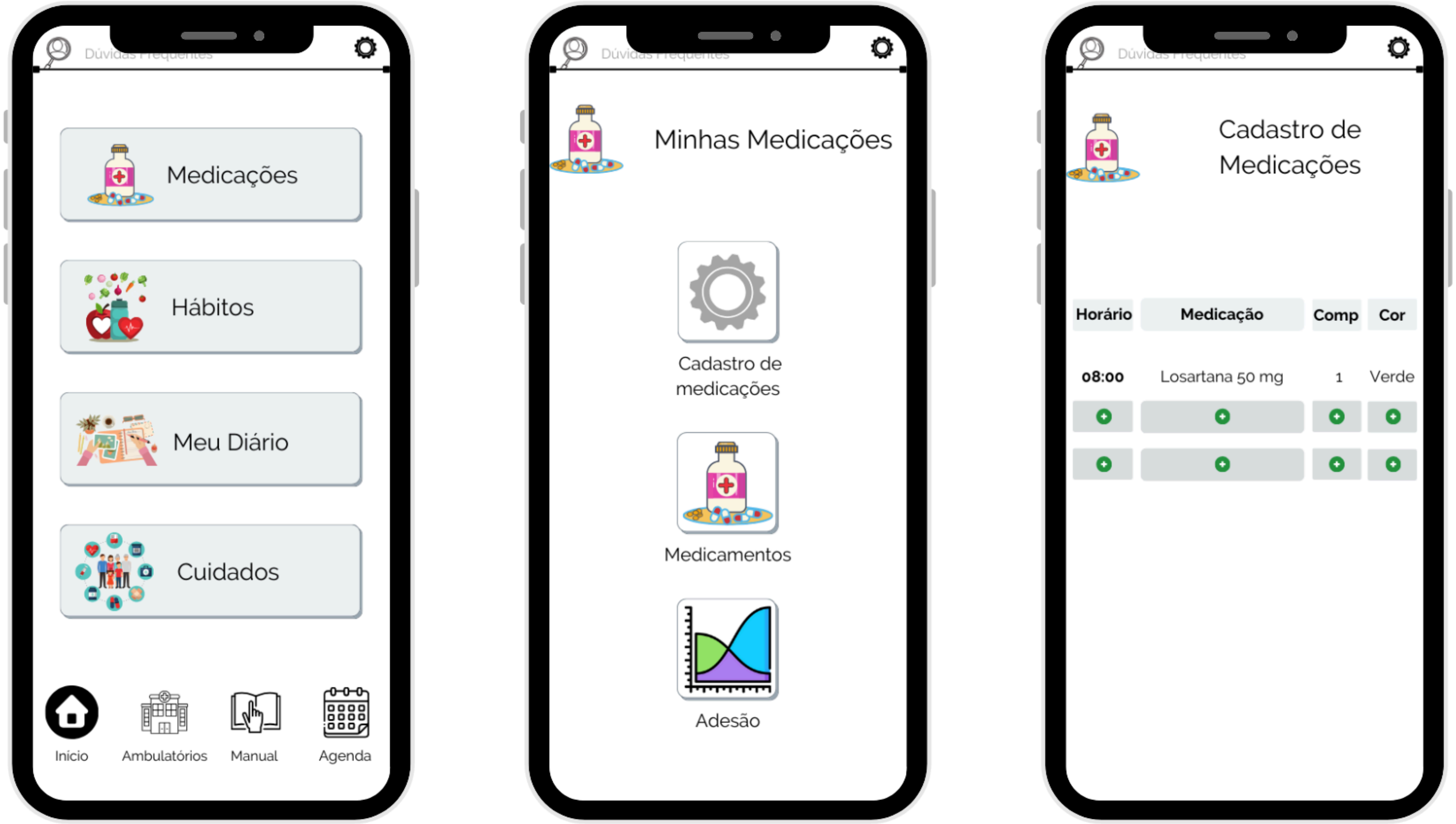

Dúvidas Frequentes
Medicações
Hábitos
Meu Diário
Cuidados
Inicio
Ambulatórios
Manual
Agenda
Dúvidas Frequentes
Minhas Medicações
Cadastro de medicações
Medicamentos
Adesão
Dúvidas Frequentes
Cadastro de Medicações
Horário
Medicação
Comp
Cor
08:00
Losartana 50 mg
1
Verde

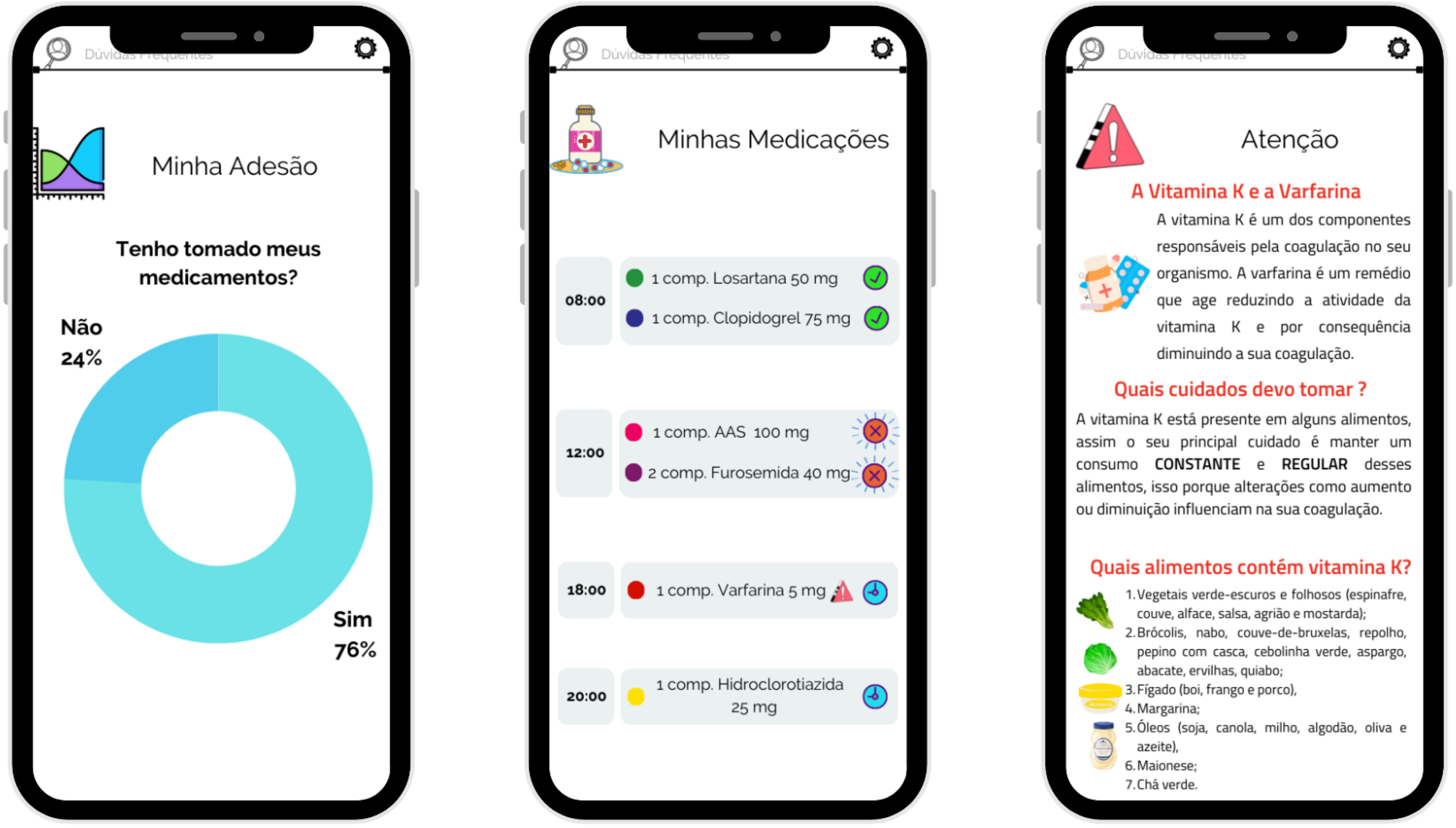
Dúvidas Frequentes
Minha Adesão
Tenho tomado meus medicamentos?
Não 24%
Sim 76%
Dúvidas Frequentes
Minhas Medicações
08:00
1 comp. Losartana 50 mg
1 comp. Clopidogrel 75 mg
12:00
1 comp. AAS 100 mg
2 comp. Furosemida 40 mg
18:00
1 comp. Varfarina 5 mg
20:00
1 comp. Hidroclorotiazida 25 mg
Dúvidas Frequentes
Atenção
A Vitamina K e a Varfarina
A vitamina K é um dos componentes responsáveis pela coagulação no seu organismo. A varfarina é um remédio que age reduzindo a atividade da vitamina K e por consequência diminuindo a sua coagulação.
Quais cuidados devo tomar ?
A vitamina K está presente em alguns alimentos, assim o seu principal cuidado é manter um consumo CONSTANTE e REGULAR desses alimentos, isso porque alterações como aumento ou diminuição influenciam na sua coagulação.
Quais alimentos contém vitamina K?
1. Vegetais verde-escuros e folhosos (espinafre, couve, alface, salsa, agrião e mostarda);
2. Brócolis, nabo, couve-de-bruxelas, repolho, pepino com casca, cebolinha verde, aspargo, abacate, ervilhas, quiabo;
3. Fígado (boi, frango e porco),
4. Margarina;
5. Óleos (soja, canola, milho, algodão, oliva e azeite),
6. Maionese;
7. Chá verde.

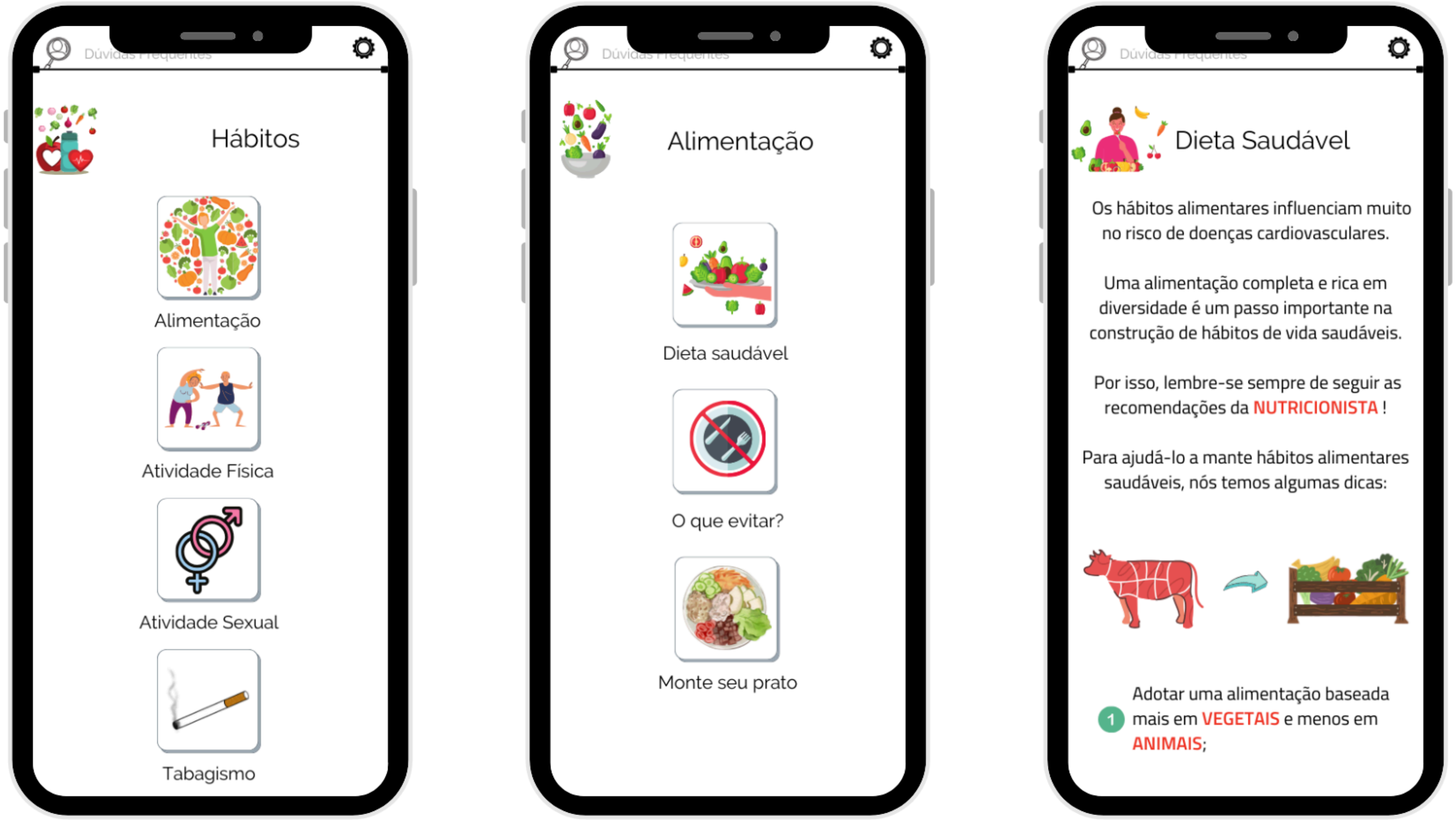
Dúvidas Frequentes
Hábitos
Alimentação
Atividade Física
Atividade Sexual
Tabagismo
Dúvidas Frequentes
Alimentação
Dieta saudável
O que evitar?
Monte seu prato
Dúvidas Frequentes
Dieta Saudável
Os hábitos alimentares influenciam muito no risco de doenças cardiovasculares.
Uma alimentação completa e rica em diversidade é um passo importante na construção de hábitos de vida saudáveis.
Por isso, lembre-se sempre de seguir as recomendações da NUTRICIONISTA !
Para ajudá-lo a mante hábitos alimentares saudáveis, nós temos algumas dicas:
1
Adotar uma alimentação baseada mais em VEGETAIS e menos em ANIMAIS;

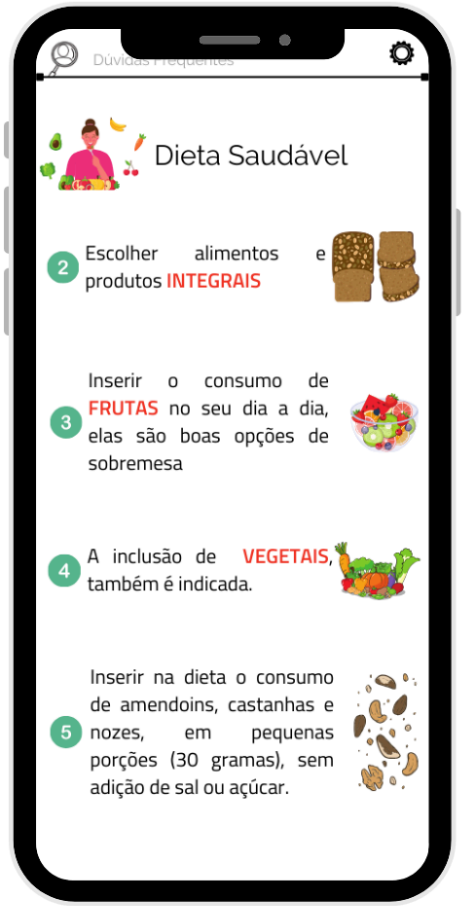
Dúvidas Frequentes
Dieta Saudável
2 Escolher alimentos e produtos INTEGRAIS
3 Inserir o consumo de FRUTAS no seu dia a dia, elas são boas opções de sobremesa
4 A inclusão de VEGETAIS, também é indicada.
5 Inserir na dieta o consumo de amendoins, castanhas e nozes, em pequenas porções (30 gramas), sem adição de sal ou açúcar.

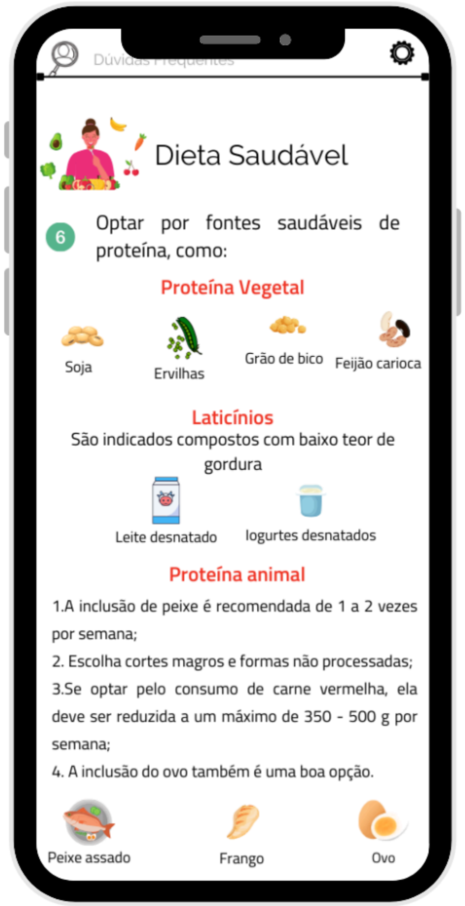
Dúvidas Frequentes
Dieta Saudável
6 Optar por fontes saudáveis de proteína, como:
Proteína Vegetal
Soja
Ervilhas
Grão de bico
Feijão carioca
Laticínios
São indicados compostos com baixo teor de gordura
Leite desnatado
Iogurtes desnatados
Proteína animal
1.A inclusão de peixe é recomendada de 1 a 2 vezes por semana;
2. Escolha cortes magros e formas não processadas;
3.Se optar pelo consumo de carne vermelha, ela deve ser reduzida a um máximo de 350 - 500 g por semana;
4. A inclusão do ovo também é uma boa opção.
Peixe assado
Frango
Ovo

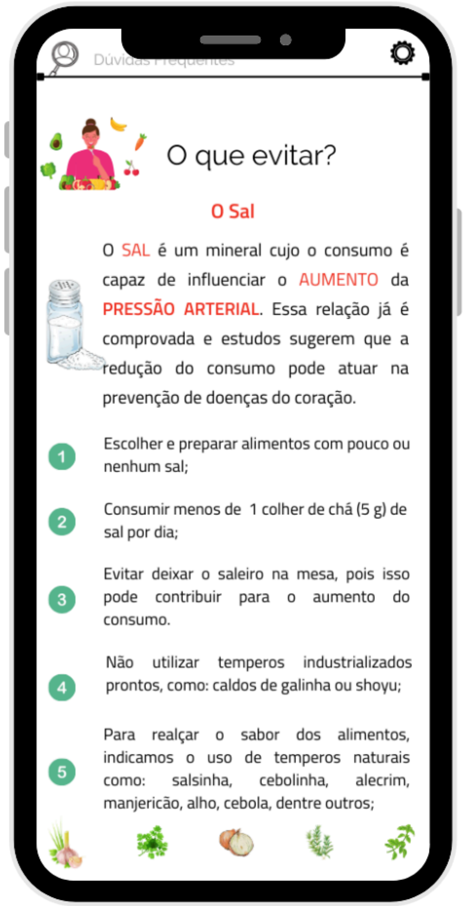
Dúvidas Frequentes
O que evitar?
O Sal
O SAL é um mineral cujo o consumo é capaz de influenciar o AUMENTO da PRESSÃO ARTERIAL. Essa relação já é comprovada e estudos sugerem que a redução do consumo pode atuar na prevenção de doenças do coração.
1 Escolher e preparar alimentos com pouco ou nenhum sal;
2 Consumir menos de 1 colher de chá (5 g) de sal por dia;
3 Evitar deixar o saleiro na mesa, pois isso pode contribuir para o aumento do consumo.
4 Não utilizar temperos industrializados prontos, como: caldos de galinha ou shoyu;
5 Para realçar o sabor dos alimentos, indicamos o uso de temperos naturais como: salsinha, cebolinha, alecrim, manjericão, alho, cebola, dentre outros;

Dúvidas Frequentes

O que evitar?

Bebidas Açucaradas

1. Refrigerantes e sucos de frutas industrializados

Alimentos processados e ultraprocessados

1. Frios, embutidos e carnes processada, como: linguiça, salsicha, bacon, presunto, apresuntado, carnes defumadas, hambúrguer etc.
2. Enlatados e conservas, com: sardinha e atum em lata, milho, ervilha, palmito em lata.

Dúvidas Frequentes

O que evitar?

Álcool

Benefícios de não consumir álcool

Sem o álcool o sistema imune volta a funcionar fortalecido.

O sono sem álcool é mais reparador

Deixar de consumir álcool pode ajudar a diminuir a sua pressão arterial e pulsação.

Você consegue perder peso com mais facilidade, isso porque além do álcool ser rico em calorias, ele pode aumentar o apetite

O consumo do álcool pode ser relacionado com mais de 60 condições médicas, incluindo: câncer, hipertensão arterial, cirrose hepática, depressão etc.

Dúvidas Frequentes

O que evitar?

Álcool

O consumo de álcool é totalmente

CONTRAINDICADO

Converse com a sua **EQUIPE** de saúde se não conseguir parar de consumir bebidas alcoólicas.

Caso opte por beber álcool as vezes, limite o consumo a um máximo de 100 g por semana.

O que são 100 g de álcool ?

Vinho — 6 taças/ semana

Cerveja — 7 latinhas/semana

Destilados — 7 doses/semana

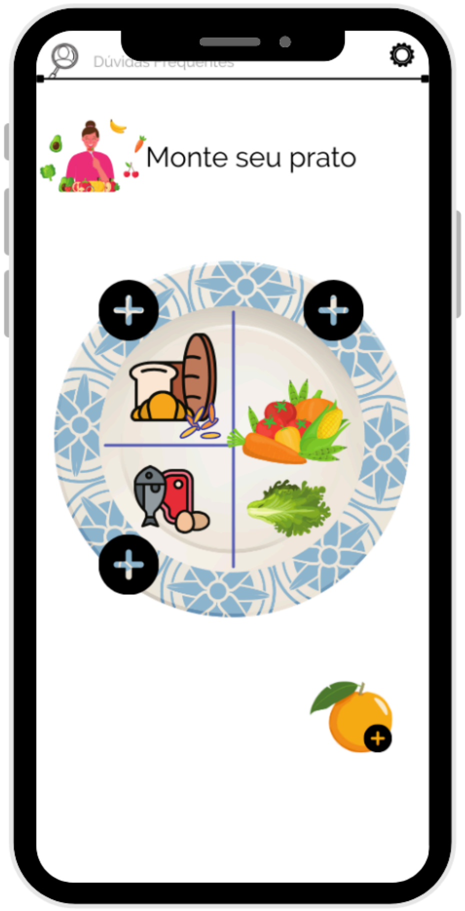
Dúvidas Frequentes
Monte seu prato

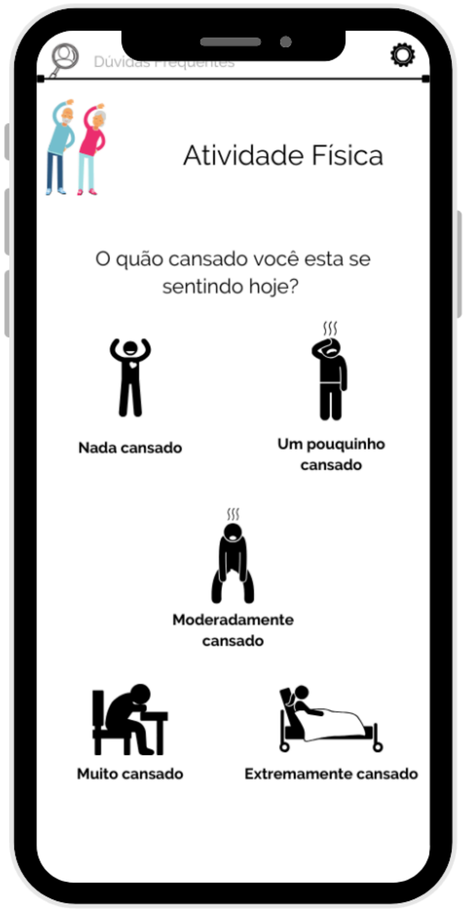
Dúvidas Frequentes
Atividade Física
O quão cansado você esta se sentindo hoje?
Nada cansado
Um pouquinho cansado
Moderadamente cansado
Muito cansado
Extremamente cansado

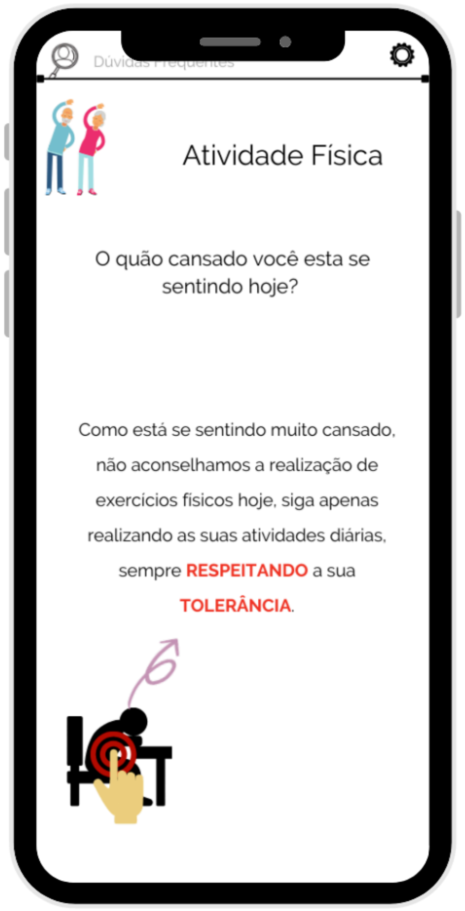
Dúvidas Frequentes
Atividade Física
O quão cansado você esta se sentindo hoje?
Como está se sentindo muito cansado, não aconselhamos a realização de exercícios físicos hoje, siga apenas realizando as suas atividades diárias, sempre RESPEITANDO a sua TOLERÂNCIA.

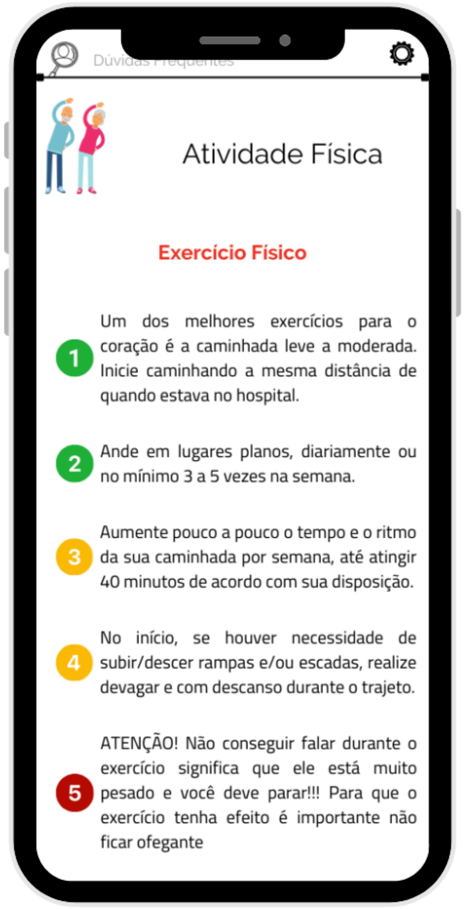
Dúvidas Frequentes
Atividade Física
Exercício Físico
1 Um dos melhores exercícios para o coração é a caminhada leve a moderada. Inicie caminhando a mesma distância de quando estava no hospital.
2 Ande em lugares planos, diariamente ou no mínimo 3 a 5 vezes na semana.
3 Aumente pouco a pouco o tempo e o ritmo da sua caminhada por semana, até atingir 40 minutos de acordo com sua disposição.
4 No início, se houver necessidade de subir/descer rampas e/ou escadas, realize devagar e com descanso durante o trajeto.
5 ATENÇÃO! Não conseguir falar durante o exercício significa que ele está muito pesado e você deve parar!!! Para que o exercício tenha efeito é importante não ficar ofegante

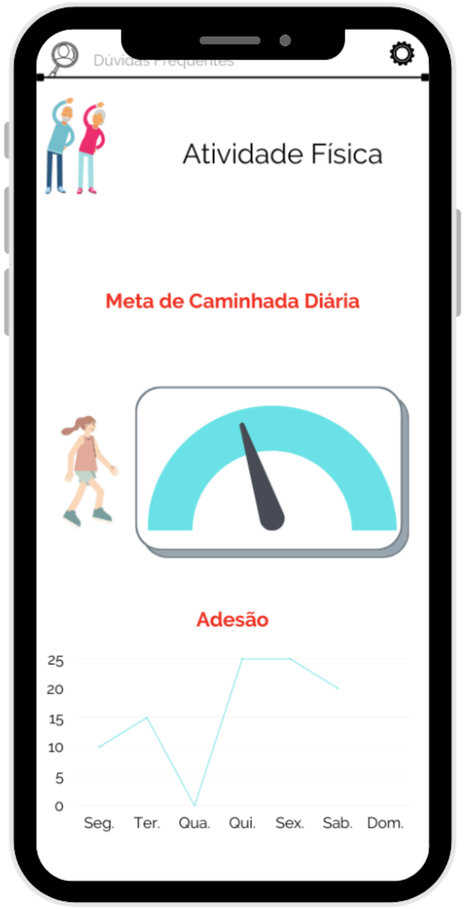
Dúvidas Frequentes
Atividade Física
Meta de Caminhada Diária
Adesão
25
20
15
10
5
0
Seg. Ter. Qua. Qui. Sex. Sab. Dom.

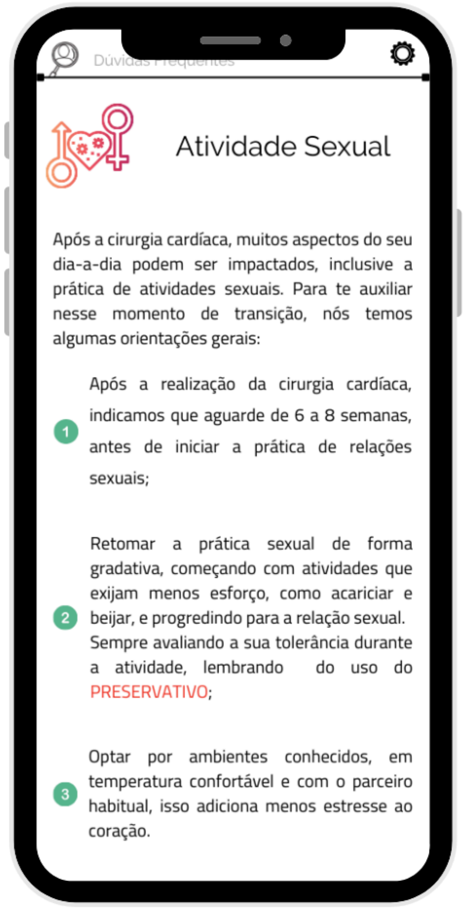
Dúvidas Frequentes
Atividade Sexual
Após a cirurgia cardíaca, muitos aspectos do seu dia-a-dia podem ser impactados, inclusive a prática de atividades sexuais. Para te auxiliar nesse momento de transição, nós temos algumas orientações gerais:
1 Após a realização da cirurgia cardíaca, indicamos que aguarde de 6 a 8 semanas, antes de iniciar a prática de relações sexuais;
2 Retomar a prática sexual de forma gradativa, começando com atividades que exijam menos esforço, como acariciar e beijar, e progredindo para a relação sexual. Sempre avaliando a sua tolerância durante a atividade, lembrando do uso do PRESERVATIVO;
3 Optar por ambientes conhecidos, em temperatura confortável e com o parceiro habitual, isso adiciona menos estresse ao coração.

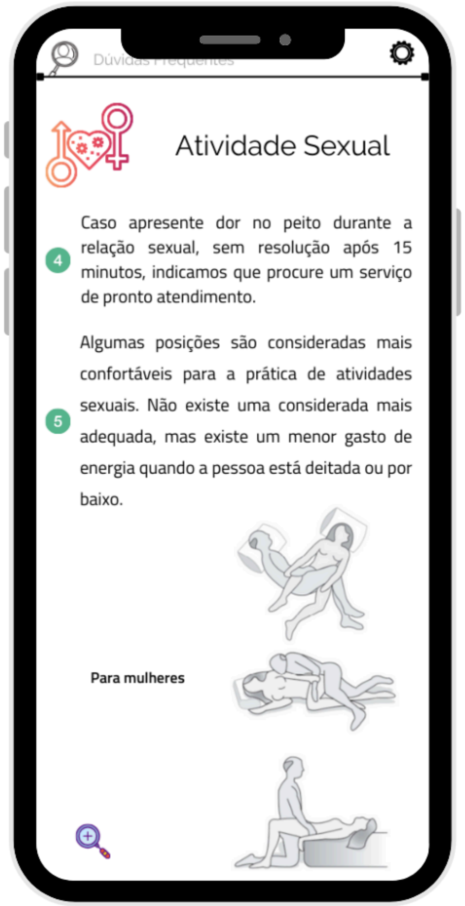
Dúvidas Frequentes
Atividade Sexual
4
Caso apresente dor no peito durante a relação sexual, sem resolução após 15 minutos, indicamos que procure um serviço de pronto atendimento.
5
Algumas posições são consideradas mais confortáveis para a prática de atividades sexuais. Não existe uma considerada mais adequada, mas existe um menor gasto de energia quando a pessoa está deitada ou por baixo.
Para mulheres

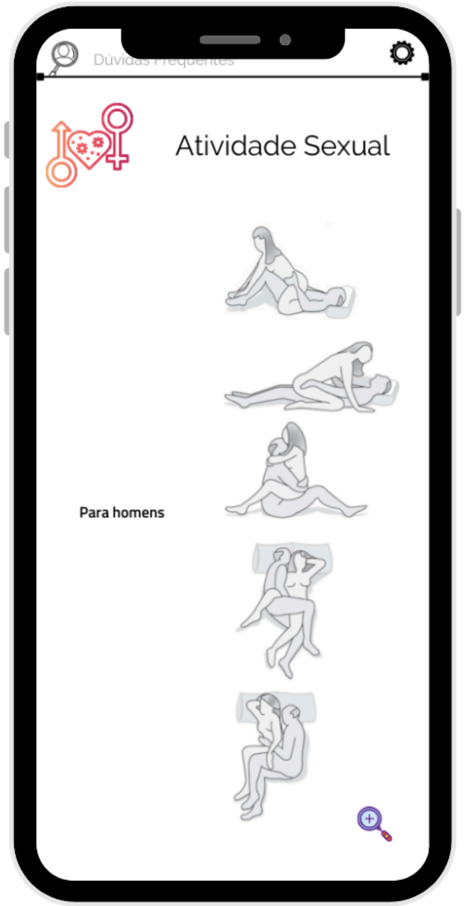
Dúvidas Frequentes
Atividade Sexual
Para homens

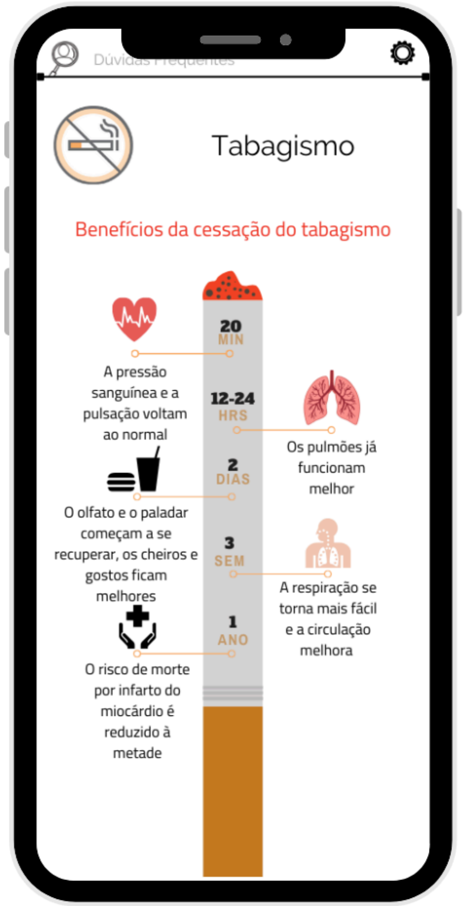
Dúvidas Frequentes
Tabagismo
Benefícios da cessação do tabagismo
20 MIN
A pressão sanguínea e a pulsação voltam ao normal
12-24 HRS
Os pulmões já funcionam melhor
2 DIAS
O olfato e o paladar começam a se recuperar, os cheiros e gostos ficam melhores
3 SEM
A respiração se torna mais fácil e a circulação melhora
1 ANO
O risco de morte por infarto do miocárdio é reduzido à metade

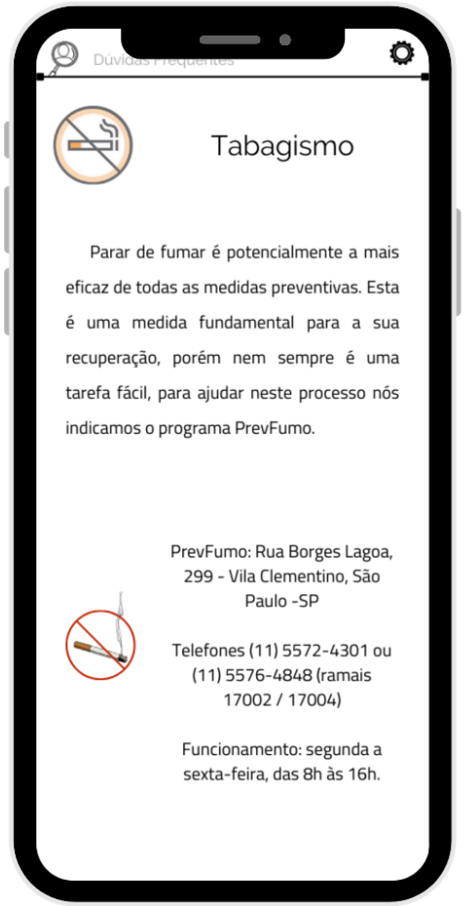
Dúvidas Frequentes
Tabagismo
Parar de fumar é potencialmente a mais eficaz de todas as medidas preventivas. Esta é uma medida fundamental para a sua recuperação, porém nem sempre é uma tarefa fácil, para ajudar neste processo nós indicamos o programa PrevFumo.
PrevFumo: Rua Borges Lagoa, 299 - Vila Clementino, São Paulo -SP
Telefones (11) 5572-4301 ou (11) 5576-4848 (ramais 17002 / 17004)
Funcionamento: segunda a sexta-feira, das 8h às 16h.

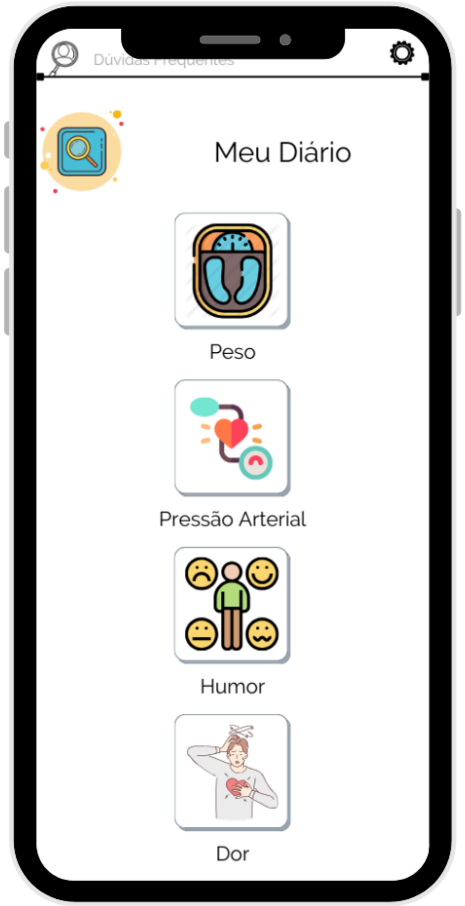
Dúvidas Frequentes
Meu Diário
Peso
Pressão Arterial
Humor
Dor

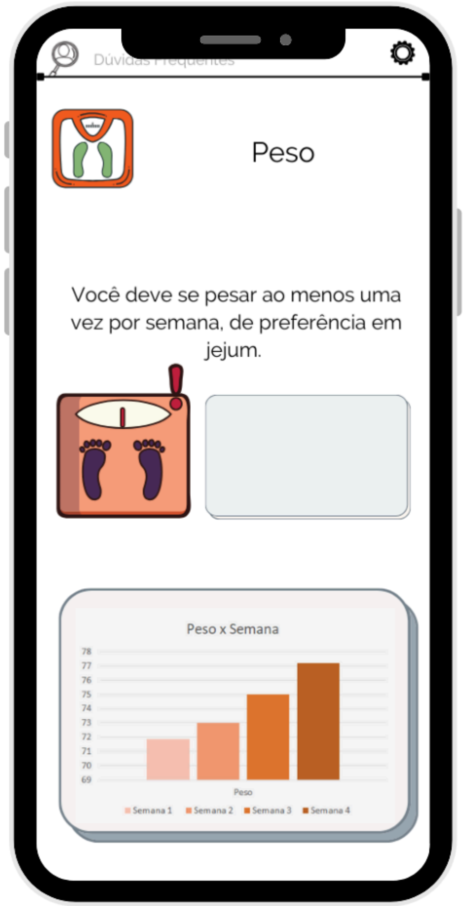
Dúvidas Frequentes
Peso
Você deve se pesar ao menos uma vez por semana, de preferência em jejum.
Peso x Semana
Peso
Semana 1
Semana 2
Semana 3
Semana 4

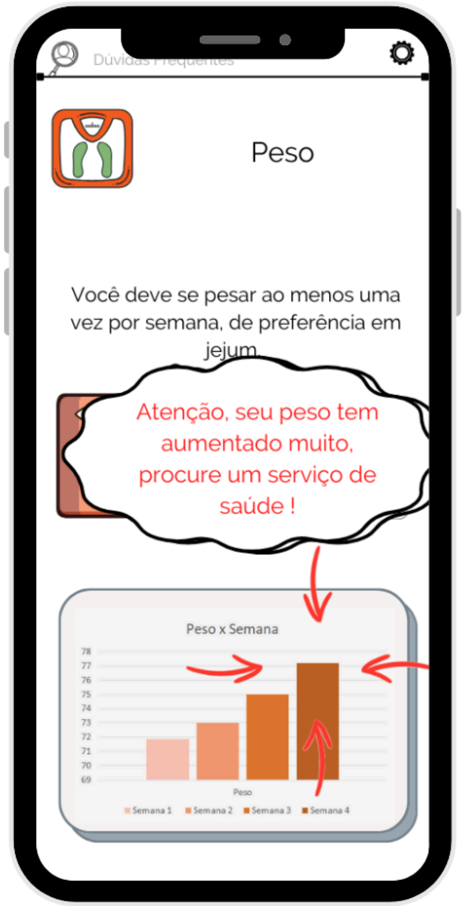
Dúvidas Frequentes
Peso
Você deve se pesar ao menos uma vez por semana, de preferência em jejum
Atenção, seu peso tem aumentado muito, procure um serviço de saúde !
Peso x Semana
Peso
Semana 1
Semana 2
Semana 3
Semana 4

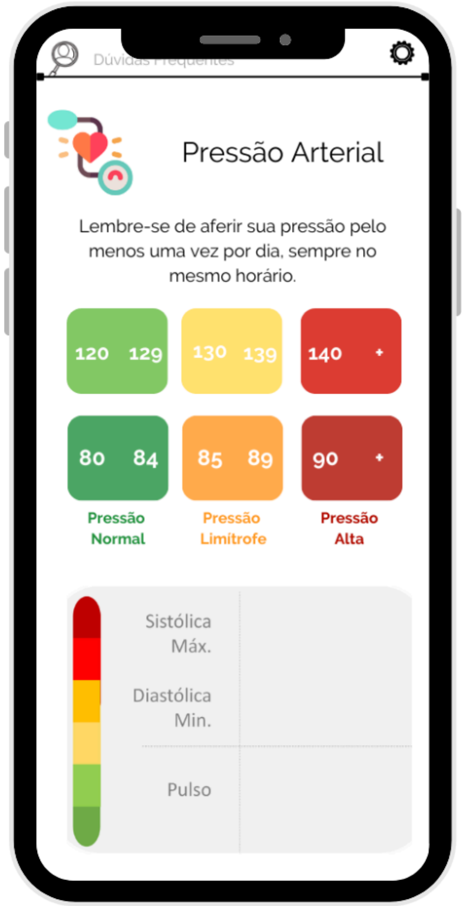
Dúvidas Frequentes
Pressão Arterial
Lembre-se de aferir sua pressão pelo menos uma vez por dia, sempre no mesmo horário.
120 129
130 139
140 +
80 84
85 89
90 +
Pressão Normal
Pressão Limítrofe
Pressão Alta
Sistólica Máx.
Diastólica Min.
Pulso

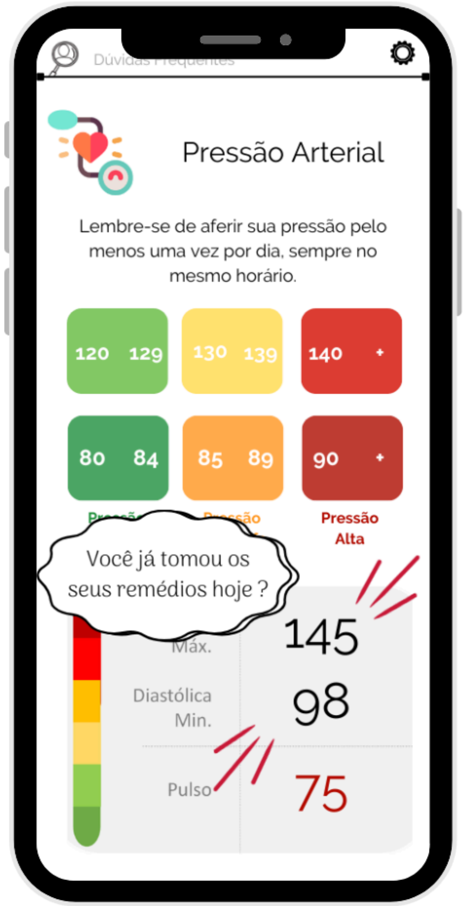
Dúvidas Frequentes
Pressão Arterial
Lembre-se de aferir sua pressão pelo menos uma vez por dia, sempre no mesmo horário.
120 129
130 139
140 +
80 84
85 89
90 +
Pressão Alta
Você já tomou os seus remédios hoje ?
Máx.
145
Diastólica Min.
98
Pulso
75

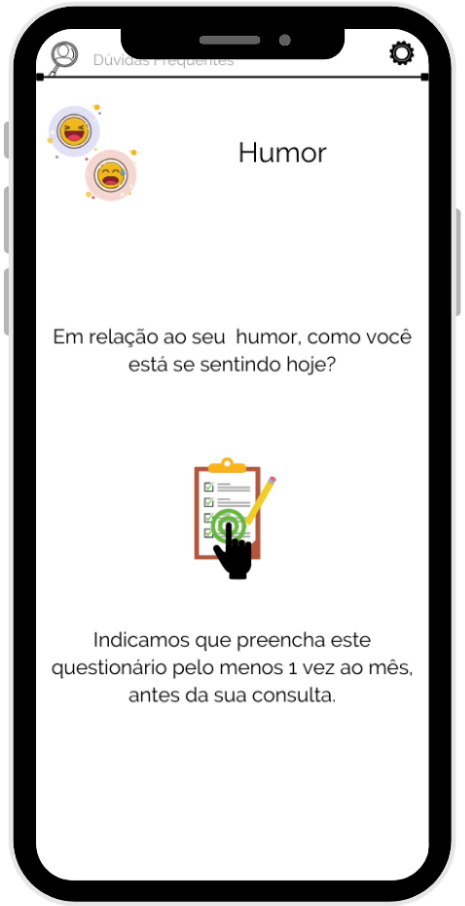
Dúvidas Frequentes
Humor
Em relação ao seu humor, como você está se sentindo hoje?
Indicamos que preencha este questionário pelo menos 1 vez ao mês, antes da sua consulta.

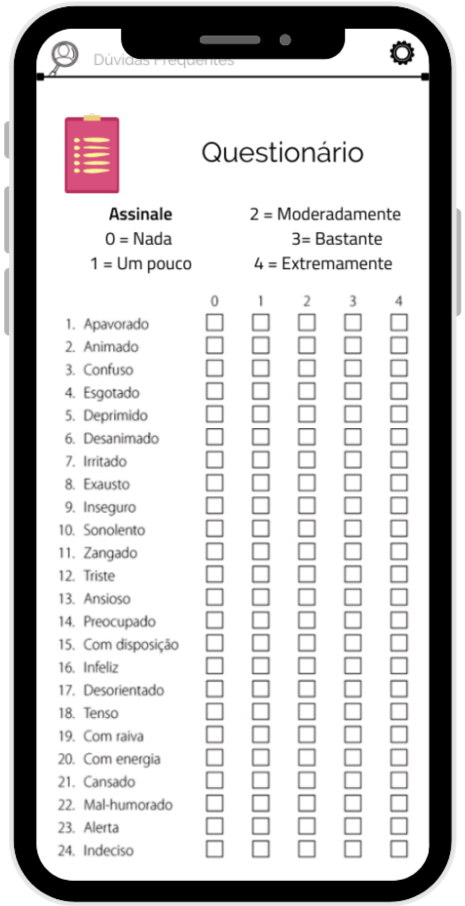
Dúvidas Frequentes
Questionário
Assinale
0 = Nada
1 = Um pouco
2 = Moderadamente
3= Bastante
4 = Extremamente
0 1 2 3 4
1. Apavorado
2. Animado
3. Confuso
4. Esgotado
5. Deprimido
6. Desanimado
7. Irritado
8. Exausto
9. Inseguro
10. Sonolento
11. Zangado
12. Triste
13. Ansioso
14. Preocupado
15. Com disposição
16. Infeliz
17. Desorientado
18. Tenso
19. Com raiva
20. Com energia
21. Cansado
22. Mal-humorado
23. Alerta
24. Indeciso

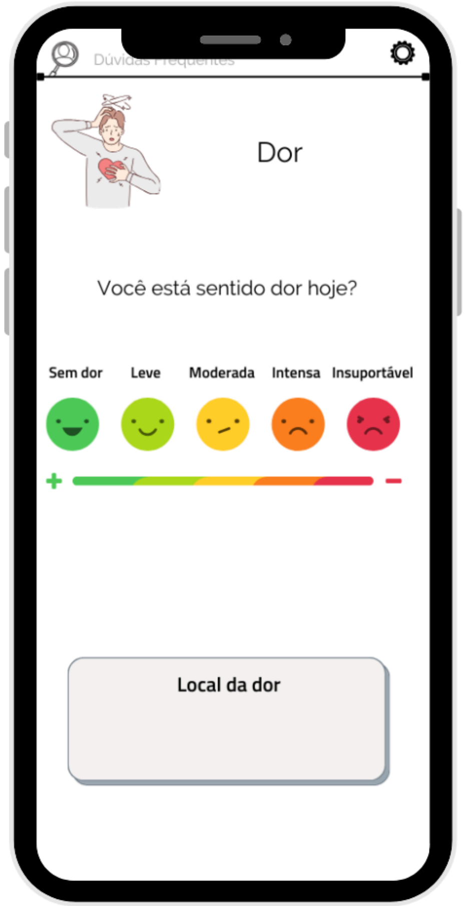
Dúvidas Frequentes
Dor
Você está sentido dor hoje?
Sem dor
Leve
Moderada
Intensa
Insuportável
+
−
Local da dor

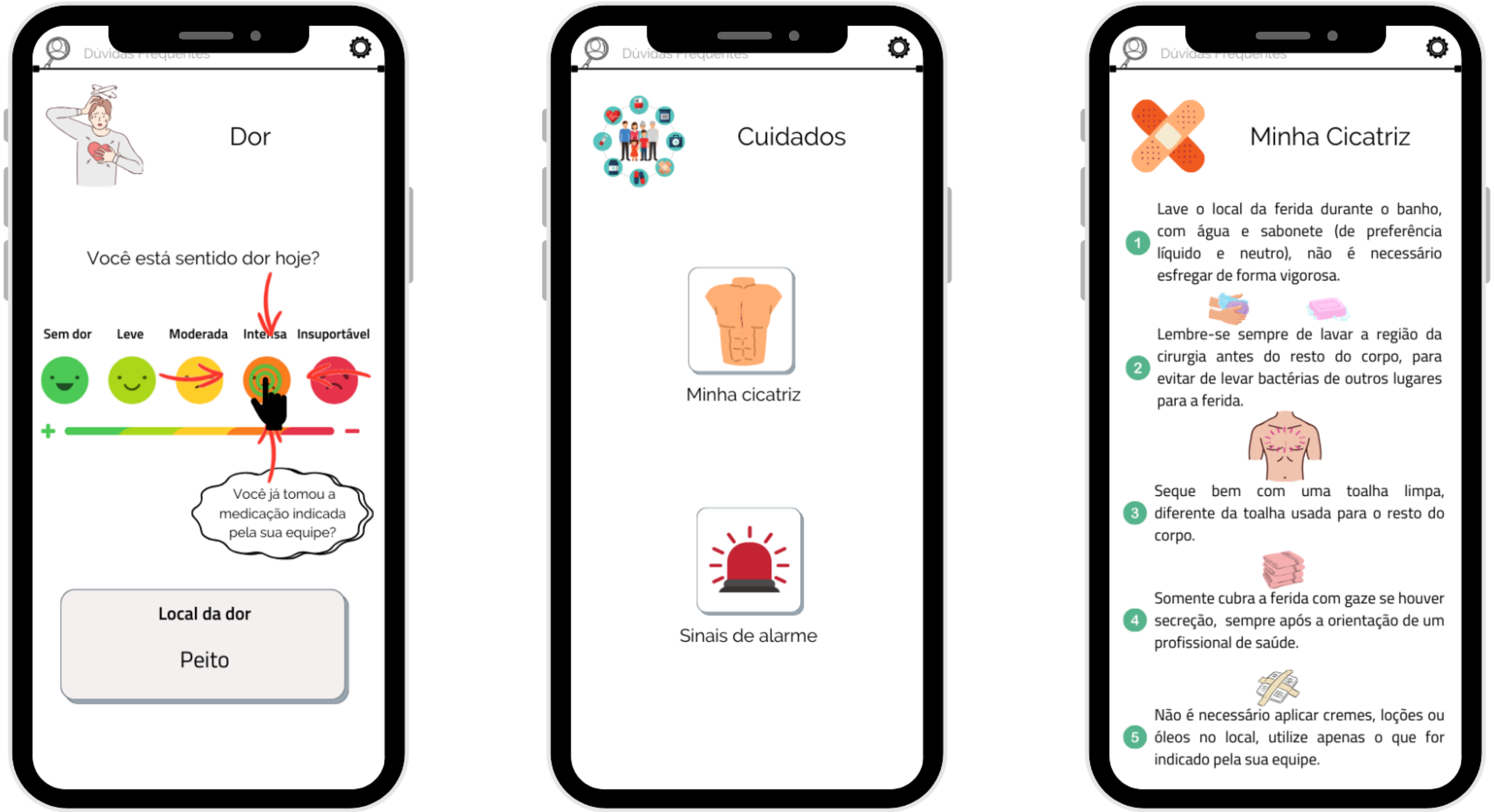
Dúvidas Frequentes
Dor
Você está sentido dor hoje?
Sem dor
Leve
Moderada
Intensa
Insuportável
+
-
Você já tomou a medicação indicada pela sua equipe?
Local da dor
Peito
Dúvidas Frequentes
Cuidados
Minha cicatriz
Sinais de alarme
Dúvidas Frequentes
Minha Cicatriz
1 Lave o local da ferida durante o banho, com água e sabonete (de preferência líquido e neutro), não é necessário esfregar de forma vigorosa.
2 Lembre-se sempre de lavar a região da cirurgia antes do resto do corpo, para evitar de levar bactérias de outros lugares para a ferida.
3 Seque bem com uma toalha limpa, diferente da toalha usada para o resto do corpo.
4 Somente cubra a ferida com gaze se houver secreção, sempre após a orientação de um profissional de saúde.
5 Não é necessário aplicar cremes, loções ou óleos no local, utilize apenas o que for indicado pela sua equipe.

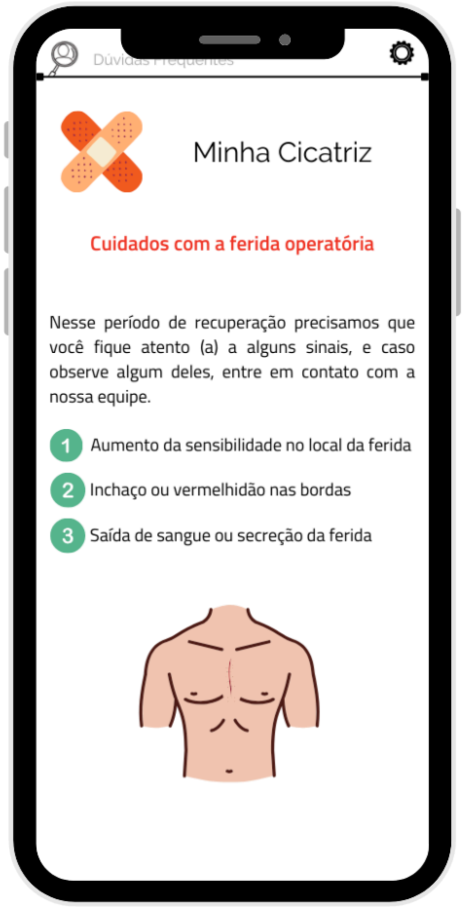
Dúvidas Frequentes
Minha Cicatriz
Cuidados com a ferida operatória
Nesse período de recuperação precisamos que você fique atento (a) a alguns sinais, e caso observe algum deles, entre em contato com a nossa equipe.
1 Aumento da sensibilidade no local da ferida
2 Inchaço ou vermelhidão nas bordas
3 Saída de sangue ou secreção da ferida

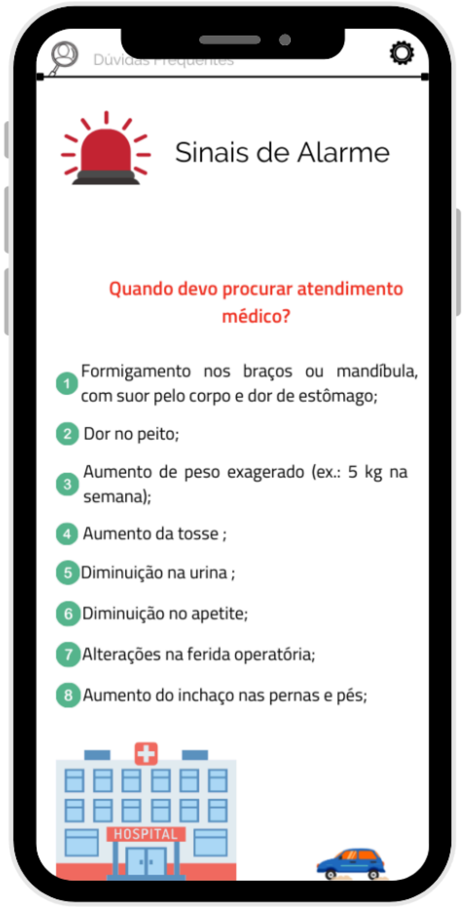
Dúvidas Frequentes
Sinais de Alarme
Quando devo procurar atendimento médico?
1 Formigamento nos braços ou mandíbula, com suor pelo corpo e dor de estômago;
2 Dor no peito;
3 Aumento de peso exagerado (ex.: 5 kg na semana);
4 Aumento da tosse ;
5 Diminuição na urina ;
6 Diminuição no apetite;
7 Alterações na ferida operatória;
8 Aumento do inchaço nas pernas e pés;
HOSPITAL

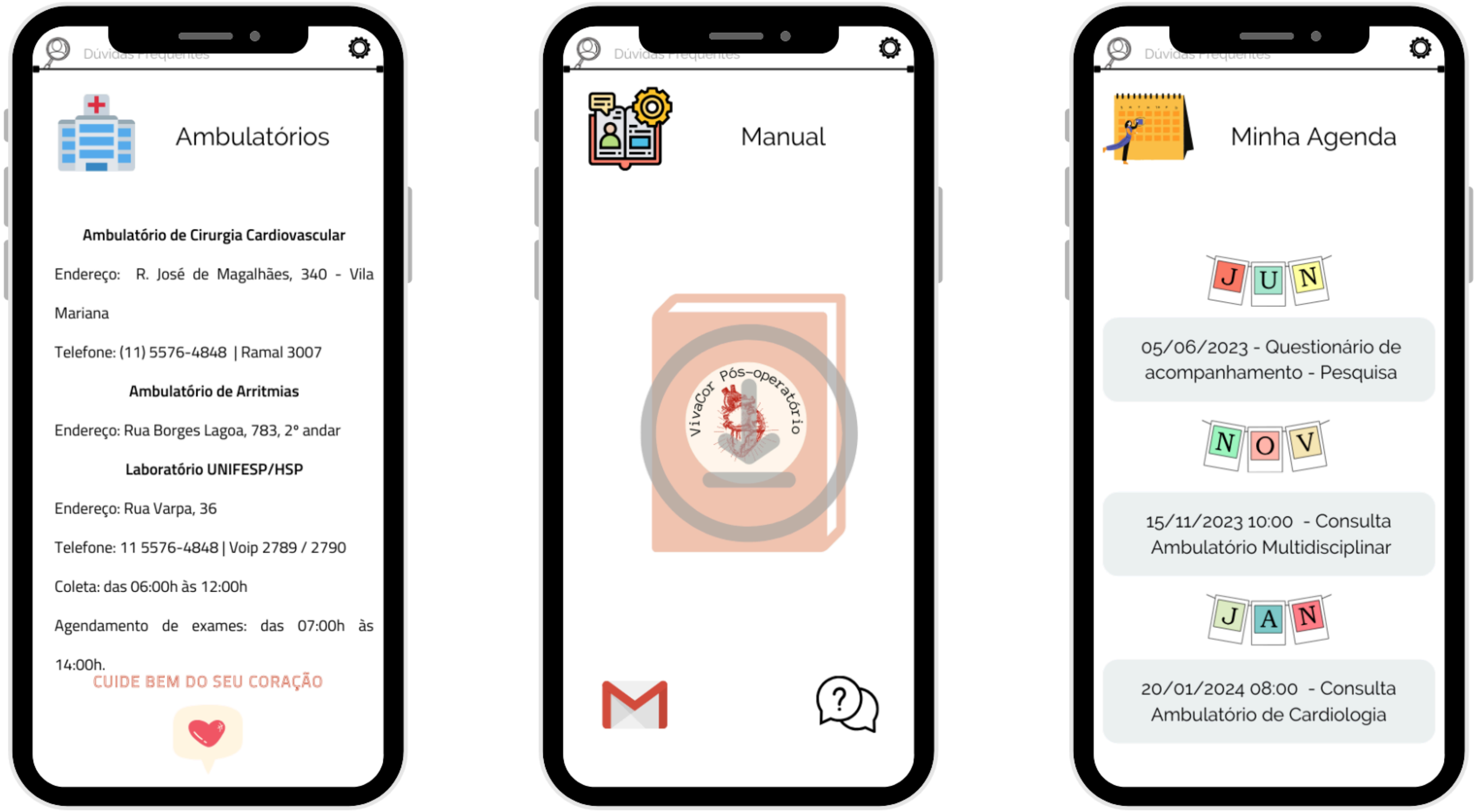
Dúvidas Frequentes
Ambulatórios
Ambulatório de Cirurgia Cardiovascular
Endereço: R. José de Magalhães, 340 - Vila Mariana
Telefone: (11) 5576-4848 | Ramal 3007
Ambulatório de Arritmias
Endereço: Rua Borges Lagoa, 783, 2º andar
Laboratório UNIFESP/HSP
Endereço: Rua Varpa, 36
Telefone: 11 5576-4848 | Voip 2789 / 2790
Coleta: das 06:00h às 12:00h
Agendamento de exames: das 07:00h às 14:00h.
CUIDE BEM DO SEU CORAÇÃO
Dúvidas Frequentes
Manual
VivaCor Pós-operatório
Dúvidas Frequentes
Minha Agenda
JUN
05/06/2023 - Questionário de acompanhamento - Pesquisa
NOV
15/11/2023 10:00 - Consulta Ambulatório Multidisciplinar
JAN
20/01/2024 08:00 - Consulta Ambulatório de Cardiologia
