# Supplementary material for: Development and validation of a mobile application prototype for postoperative cardiac surgery
Source: Rev Bras Enferm. 2024 Oct 7;77(5):e20230491. doi: 10.1590/0034-7167-2023-0491 (PMC11458145; doi:10.1590/0034-7167-2023-0491)
Supplement: Translation

# Translation: Desenvolvimento e validação do protótipo de um aplicativo móvel para pós-operatório de cirurgia cardíaca

**DOI:** 10.1590/0034-7167-2023-0491pt

**Authors:** Gabriele Cardoso Gonçalves Alves, Fabiola Leticia Damascena Amador, Vagner Rogério dos Santos, Rita Simone Lopes Moreira

**Affiliations:** I Universidade Federal de São Paulo. São Paulo, São Paulo, Brasil

**Keywords:** Aplicativos Móveis, Doenças Cardiovasculares, Cuidados Pós-Operatórios, Autocuidado, Estudo de Validação

## Abstract

**Objetivos:**

desenvolver e validar os conteúdos, a aparência e a semântica do protótipo de um aplicativo para acompanhamento de pacientes em pós-operatório de cirurgia cardíaca.

**Métodos:**

estudo de desenvolvimento tecnológico baseado no *Design* Instrucional Contextualizado. A avaliação de conteúdo e aparência foi realizada por um comitê de especialistas, e a validação semântica foi realizada por pacientes do ambulatório de cirurgia cardíaca.

**Resultados:**

o protótipo do aplicativo resultou em 43 telas, validadas por 17 especialistas em saúde, com resultados de razão de validade de conteúdo e índice de validade de aparência totais de 0,86 e 0,99, respectivamente. Em relação à validação semântica, 10 pacientes participaram da coleta de dados, com índice de validade de conteúdo total de 0,978.

**Conclusões:**

o protótipo do aplicativo “VivaCor PósOp” apresentou evidências de validade de conteúdo, aparência e semântica, com potencial para estimular o autocuidado em pacientes em pósoperatório de cirurgia cardíaca.

## INTRODUÇÃO

Com o avanço das tecnologias e o envelhecimento da população mundial, o uso de estratégias de saúde digital tem se tornado uma necessidade cada vez mais crucial no contexto global^(1)^. Sendo assim, a crescente prevalência de doenças crônicas não transmissíveis (DCNT), aliada à busca por recursos que promovam o autocuidado e utilizem a tecnologia como ferramenta no setor de saúde, tem impulsionado e fortalecido ações no campo da saúde digital^(1)^.

De forma a aprimorar a oferta de cuidados em saúde e superar algumas das barreiras dos sistemas de saúde globais, o emprego de tecnologias digitais, como *a eHealth,* que se baseia no uso de tecnologias de informação e comunicação (TIC) aplicadas à saúde, e a *mHealth,* que aborda o uso de TICs apoiadas por dispositivos móveis, tem se destacado como estratégias promissoras^(2, 3, 4)^.

Neste cenário, a integração das TICs na área da saúde passou a ser considerada fundamental para enfrentar o desafio das DCNT e das multimorbidades^(1)^. Dentre as DCNT, as doenças cardiovasculares (DCVs) constituem a principal causa de morte no mundo, correspondendo a aproximadamente 17,6 milhões de mortes no ano de 2016^(5)^. Em relação aos procedimentos cirúrgicos por DCVs, a Sociedade Brasileira de Cardiologia (SBC) relatou um aumento de 64% entre 2008 e 2019, o que representa um grande impacto tanto na perspectiva de custos quanto no comprometimento do estado de saúde do indivíduo^(6)^.

Considerando os períodos cirúrgicos, o pós-operatório é um momento complexo e extremamente delicado, principalmente em cirurgias de grande porte, nas quais frequentemente são observados incidentes como dor, eventos trombóticos, fadiga, convalescença prolongada, dentre outros^(7)^. Isto posto, para auxiliar a recuperação do paciente, programas de reabilitação cardíaca (RC) têm sido cada vez mais empregados^(8)^. A RC é uma intervenção baseada na atuação da equipe multiprofissional, que tem como objetivo reduzir os reflexos fisiológicos e psicológicos das DCVs, além de auxiliar na estabilização clínica do paciente^(8)^.

Nesse sentido, os programas de RC têm como elemento-chave estratégias de envolvimento baseadas no autocuidado e autogerenciamento do paciente^(8)^. Com foco no autocuidado de pacientes com DCVs, o uso de aplicativos móveis tem sido uma tendência crescente em todo o mundo^(3)^. Assim, o uso de estratégias de *eHealth* e *mHealth* são consideradas alternativas inovadoras em relação aos métodos tradicionais de educação em saúde para a entrega das informações de forma acessível^(^9-10^)^. Diante disso, a utilização de aplicativos móveis para estruturar o cuidado centrado no paciente mostra-se uma abordagem relevante, fornecendo informações essenciais sobre a doença durante o processo de reabilitação^(3)^.

Entretanto, mesmo diante disso, ao realizar uma busca por aplicativos nas lojas virtuais *Google” Play Store* e *Apple” Store,* não foram encontrados recursos direcionados a pacientes em pós-operatório de cirurgia cardíaca no idioma português. Portanto, considerando o progressivo aumento do uso da internet, principalmente nas faixas etárias acima de 45 anos^(2)^, optou-se pelo desenvolvimento de um aplicativo para acompanhamento de pacientes em pós-operatório de cirurgia cardíaca, com o propósito de prover uma ferramenta capaz de armazenar a história clínica do paciente, e consequentemente reduzir a desinformação, reinternações recorrentes, com menor impacto nos custos de saúde.

## OBJETIVOS

Desenvolver e validar os conteúdos, a aparência e a semântica do protótipo de um aplicativo para acompanhamento de pacientes em pós-operatório de cirurgia cardíaca.

## MÉTODOS

### Aspectos éticos

O protocolo de pesquisa, originado de uma dissertação de mestrado, foi apreciado e aprovado pelo Comitê de Ética em Pesquisa com Seres Humanos de uma universidade federal em São Paulo.

### Desenho, período e local de estudo

Estudo de desenvolvimento tecnológico e de validação, baseado no *Design* Instrucional Contextualizado^(11)^, direcionado para a criação e análise de evidências de validade de conteúdo, aparência e semântica do protótipo de um aplicativo móvel. Foi realizado em ambiente virtual com jurados especialistas entre outubro de 2022 e março de 2023 e no ambulatório de cirurgia cardíaca de uma universidade federal em São Paulo, entre março e abril de 2023, com pacientes. Os dados analisados neste estudo são oriundos de uma dissertação de mestrado, norteados pela ferramenta *Guideline for Reporting for Intervention Development Studies* (GUIDED)^(12)^.

### População, critérios de inclusão e exclusão

Para a etapa de validação de conteúdos e aparência do protótipo do aplicativo, foram convidados 200 profissionais da saúde (enfermeiros, fisioterapeutas, nutricionistas, farmacêuticos e médicos), sendo 40 de cada área. O recrutamento foi precedido de caracterização do perfil, avaliando a área de atuação, tempo de experiência profissional e titulação acadêmica, considerando uma pontuação mínima de cinco nos critérios adaptados do Sistema de Classificação de Especialistas do Modelo de Validação Fehring^(13, 14)^.

Os profissionais selecionados receberam uma carta convite via e-mail, enviada por meio da plataforma *RedCap*®, com todas as informações sobre o estudo, incluindo seus objetivos, público-alvo, questionários a serem respondidos e instituição de vínculo, além do Registro de Consentimento Livre e Esclarecido (RCLE). Uma vez aceito o convite, os jurados receberam um segundo e-mail contendo os questionários e um link para acesso ao *Google Drive*®, onde encontraram o *mockup* em vídeo (.mp4) e uma versão em *Portable Document Format* (PDF) com as telas do aplicativo.

Após o recrutamento, foram excluídos jurados que não atingiram a pontuação mínima de cinco no Sistema de Classificação de Especialistas do Modelo de Validação Fehring^(13, 14)^, e os especialistas que não completaram qualquer um dos questionários enviados dentro do período de quatro meses, após envios de até três lembretes de participação. Também foram excluídos aqueles que não justificaram suas avaliações e os que expressaram dúvidas importantes em relação à população ou tecnologia avaliada em seus comentários, comprometendo o seu julgamento.

Na fase de validação semântica, foram incluídos os pacientes que estavam em pós-operatório mediato ou tardio de cirurgia cardíaca realizada no hospital de referência de uma universidade federal em São Paulo, que seguiam em acompanhamento no ambulatório da mesma instituição, maiores de 18 anos, alfabetizados e que aceitaram participar do estudo a partir da assinatura do Termo de Consentimento Livre e Esclarecido (TCLE). Pacientes com deficiência visual ou com quaisquer limitações que pudessem comprometer a avaliação das telas foram excluídos do estudo.

### Protocolo do estudo

A metodologia adotada para o desenvolvimento tecnológico baseou-se no conceito de *Design* Instrucional Contextualizado (DIC)^(11)^. O processo metodológico adotado consistiu em quatro fases interligadas que foram executadas de forma cíclica^(11)^, sendo composto pelas seguintes etapas: análise, *design* e desenvolvimento, implementação e avaliação, conforme observado na Figura 1.

**Figura 1:** Etapas do estudo segundo a metodologia *Design* Instrucional Contextualizado

#### Fase 1: Análise

O protótipo do aplicativo foi desenvolvido no contexto de um grupo de pesquisa, composto por profissionais de saúde da equipe multidisciplinar e profissionais da área de tecnologia. Na etapa de análise, os pesquisadores caracterizaram a população, optando por um aplicativo direcionado a pacientes em pós-operatório mediato ou tardio de cirurgia cardíaca, e também consideraram importante a atualização dos dados do manual de alta da instituição após a cirurgia cardíaca.

#### Fase 2: Design e Desenvolvimento

Os conteúdos do protótipo do aplicativo foram desenvolvidos com base em uma revisão integrativa de literatura realizada pelos autores em 2022 para este propósito, sendo incluídos artigos, diretrizes e recomendações relacionadas à RC, à prevenção de DCV e ao pós-operatório de cirurgia cardíaca, publicadas nos últimos 10 anos. A partir desta busca, foram elencados os principais eixos de atuação, os quais constituíram os seguintes ícones do protótipo do aplicativo: medicamentos, hábitos, meu diário e cuidados.

Posteriormente, o protótipo do aplicativo foi estruturado por meio da plataforma Canva®. O modelo inicial, desenvolvido pelo autor em parceria com um segundo revisor, foi apresentado durante reuniões de um grupo de pesquisa. Após duas apresentações, chegou-se ao modelo final, composto por 43 telas.

#### Fase 3: Implementação

A etapa de implementação envolveu a avaliação de conteúdo e aparência por um comitê de especialistas^(15)^ e a validação semântica por um grupo piloto de pacientes. A validação consiste na verificação da presença do atributo de validade, ou seja, a partir desse processo, a tecnologia é considerada adequada para cumprir a tarefa à qual se propõe^(15)^.

Uma vez que não havia um teste estatístico específico para avaliar a validade do conteúdo, foi realizada uma abordagem qualitativa, com um comitê de especialistas^(16)^ composto por um número de jurados variando de seis a vinte, de acordo com estudos anteriores^(17)^. O número de profissionais no comitê foi determinado com base na razão de validade de conteúdo (RVC) de Lawshe, que mede a concordância dos especialistas quanto à essencialidade dos itens^(18)^.

A validação do conteúdo foi realizada por meio do Instrumento de Validação de Conteúdo Educativo em Saúde (IVCES)^(19)^. As respostas no IVCES foram equiparadas com as categorias propostas por Lawshe: “não necessário”, “útil, mas não essencial” e “essencial”^(18)^. O cálculo do RVC foi realizado para cada item e domínio separadamente.

Já a validação de aparência foi conduzida com o Instrumento de Validação de Aparência de Tecnologia Educacional em Saúde (IVATES) ^(20)^. O índice de validade de aparência (IVA) foi calculado para cada um dos doze itens no IVATES e para o questionário como um todo. O valor mínimo de concordância considerado aceitável foi 0,78 para que o item não necessitasse de revisão^(20)^.

A validação semântica desempenha um papel crucial na avaliação de tecnologias educacionais, permitindo ajustes no layout e conteúdo, alinhando-se com a metodologia DIC^(11)^. Essa análise envolve o público-alvo, com especial atenção aos estratos sociais menos favorecidos, por meio de um pré-teste, garantindo que os itens sejam compreensíveis e adequados à população-alvo da tecnologia educacional^(21, 22)^. A coleta de dados envolveu dois questionários: o primeiro abordou aspectos sociodemográficos e de identificação, enquanto o segundo, adaptado do estudo de Oliveira^(23)^, consistiu em cinco eixos, estruturados com base no *Suitability Assessment of Materials* (SAM)^(24)^. O Instrumento de Validação Semântica (IVS), um questionário adaptado, utiliza uma escala Likert de quatro pontos para avaliar itens. A confiabilidade do IVS adaptado foi avaliada pelo coeficiente ômega (to) de McDonald, cujos valores de to > 0,70 indicam confiabilidade do conjunto de fatores^(25)^.

Para a avaliação dos resultados do questionário de validação semântica, foi utilizado o Índice de Validade de Conteúdo (IVC)^(17)^, que retrata a proporção de avaliadores em concordância com os itens do questionário, calculado tanto individualmente para cada item quanto para os eixos temáticos e para o instrumento como um todo.

#### Fase 4: Avaliação

A fase de avaliação contou com a análise dos resultados obtidos na fase de implementação. A coleta de dados foi realizada por meio da plataforma *RedCap®* tanto para os jurados quanto para os pacientes, e os dados foram armazenados no servidor e, posteriormente, recuperados em planilhas do *Microsoft Excel®.* Para a validação, a partir da contagem das pontuações dos questionários IVCES, IVATES e IVS-Adaptado, foi realizada uma análise estatística simples com o cálculo do RVC, IVA, IVC e w de McDonald. Para a presente análise de dados, a base teórica utilizada foi descrita por Field^(25)^, sendo o valor de significância estatística igual a 5% (p ≤ 0,05), com o uso do *software SPSS Statistics,* versão 28.0 (IBM Corp., Armonk, NY, EUA).

## RESULTADOS

A partir de uma revisão integrativa de literatura foram identificadas quatro diretrizes, sendo uma da SBC^(26)^, uma da *American Heart Association* (AHA)^(27)^ e duas da *European Society of Cardiology* (ESC)^(28, 29)^, direcionadas para RC, prevenção de DCVs e pós-operatório de doenças cardíacas. Adicionalmente, foram incluídos quatro artigos de revisão que abordaram a RC^(8, 30, 31, 32)^, além de uma recomendação da AHA^(33)^. A partir dos achados, foram construídos os ícones do protótipo englobando os seguintes tópicos: cuidados com a ferida operatória, medicamentos, atividade física, relações sexuais, alimentação, tabagismo e etilismo, controle de pressão arterial, peso e sinais de alarme para procura de atendimento médico imediato. A Figura 2 representa as telas principais do protótipo do aplicativo.

**Figura 2:** Telas do protótipo do aplicativo VivaCor PósOp

Para a etapa de validação por especialistas, dos 200 jurados convidados, 36 aceitaram contribuir com o estudo, e destes, apenas 27 completaram todos os questionários propostos. Para garantir a qualidade da amostra, foram excluídas as respostas dos profissionais que pontuaram cinco ou menos no Sistema de Classificação de Especialistas do Modelo de Validação Fehring^(13)^ (n = 6), bem como dos jurados que deixaram claro, em seus comentários, que não entenderam a proposta de avaliação, o público-alvo ou o questionário (n = 4).

A amostra foi integrada por 17 jurados, sendo a maioria enfermeiros (n = 10), seguidos de fisioterapeutas (n = 4), nutricionista (n = 1) e farmacêutico (n = 1). Todos os participantes tinham expertise em cardiologia, sendo seis doutores, seis mestres, quatro pós-doutores e um especialista. Os escores segundo critérios de Fehring variaram de seis a 13 pontos, sendo a média de 10,7 pontos. Em relação à idade dos especialistas, a média foi de 36 anos, a mediana foi de 40,4 anos e os valores mínimo e máximo foram de 28 e 63 anos, respectivamente. Esta caracterização detalhada pode ser consultada no Material Suplementar.

Considerando a relação entre RVC e número de especialistas avaliadores estabelecida por Lawshe, a fim de garantir que é improvável que a concordância seja devida ao acaso, identificou-se que o valor mínimo de RVC era de 0,49^(18)^. Os resultados de RVC por eixo foram: Objetivos = 0,86; Estrutura = 0,82; Relevância = 1. Em relação à avaliação do RVC por item, observou-se uma variação entre 0,53 a 1. O detalhamento das pontuações pode ser consultado no Material Suplementar.

Os jurados foram convidados a fornecer comentários sobre questões em que havia discordância parcial ou total, além de terem a oportunidade de fazer comentários gerais ao final do questionário. Todos os comentários pertinentes foram considerados e discutidos em um grupo de pesquisa, e as telas foram revisadas pela autora após as modificações. Posteriormente, dois jurados independentes avaliaram os tópicos alterados e procuraram possíveis erros ortográficos. Além da validação de conteúdo, os especialistas também validaram a aparência da interface do protótipo usando o IVATES, calculando o IVA, que variou de 0,94 a 1 (Tabela 1). Da mesma forma, os jurados foram convidados a comentar as alternativas para as quais referiam “discordar parcialmente”, “discordar” ou “discordar totalmente”. Assim, para este questionário obtivemos apenas duas sugestões, sendo elas: (i) a separação das seções “humor” e “dor”; (ii) o aumento do tamanho das figuras no campo “atividade sexual”. Ambas as sugestões foram aceitas e o layout foi alterado.

**Tabela 1:** Índice de Validade de Aparência das telas do aplicativo utilizando o Instrumento de Validação de Aparência de Tecnologia Educacional em Saúde, São Paulo, São Paulo, Brasil, 2024

Item do IVATES	IVA-I	IVA-T
1. As ilustrações estão adequadas para o público-alvo	1	
2. As ilustrações são claras e transmitem facilidade de compreensão	0,941	
3. As ilustrações são relevantes para compreensão do conteúdo pelo público-alvo	1	
4. As cores das ilustrações estão adequadas para o tipo de material	1	
5. As formas das ilustrações estão adequadas para o tipo de material	1	
6. As ilustrações retratam o cotidiano do público-alvo da intervenção	1	
7. A disposição das figuras está em harmonia com o texto	1	
8. As figuras utilizadas elucidam o conteúdo do material educativo	1	
9. As ilustrações ajudam na exposição da temática e estão em uma sequência lógica	1	
10. As ilustrações estão em quantidade adequadas no material educativo	1	
11. As ilustrações estão em tamanhos adequados no material educativo	0,941	
12. As ilustrações ajudam na mudança de comportamentos e atitudes do público-alvo	1	0,990

*IVATES – Instrumento de Validação de Aparência de Tecnologia Educacional em Saúde; IVA-I – Índice de validade de aparência do item; IVA-T – Índice de validade de aparência total.*

O estudo abordou 12 pacientes elegíveis em um ambulatório de cirurgia cardíaca, coletando dados em duas ocasiões distintas, com o aceite de 10 pacientes. A maioria dos participantes era do sexo masculino (90%), com 40% tendo ensino fundamental incompleto e uma idade média de 66,07 anos. Houve uma alta prevalência de comorbidades autorreferidas, destacando-se hipertensão arterial sistêmica (90%), dislipidemia (70%) e insuficiência cardíaca (40%). Em relação à cirurgia cardíaca, 30% dos pacientes haviam realizado a intervenção há 1-2 anos, 20% há 3-4 anos, e os demais haviam passado mais de cinco anos desde a intervenção (50%). A caracterização detalhada dos pacientes está disponível no Material Suplementar.

Precedente à validação semântica, foi analisada a consistência interna dos domínios do instrumento usando o coeficiente ômega de McDonald, como exposto na Tabela 2. Em relação à validação semântica, nenhum item foi considerado inadequado, mas três perguntas foram classificadas como parcialmente adequadas. Os resultados do IVC mostraram uma alta pontuação geral (0,978). O detalhamento das avaliações pode ser consultado no Material Suplementar.

**Tabela 2:** Índice de Validade de Conteúdo e Ômega de McDonald do Instrumento de Validação Semântica Adaptado, São Paulo, São Paulo, Brasil, 2024

Domínio do Instrumento de Validação Semântica	IVC	ω
Objetivos	0,967	0,971
Organização	0,980	0,837
Linguagem	1	0,892
Aparência	1	0,821
Motivação	0,933	0,687

*IVC – Índice de Validade de Conteúdo; ω – ômega de McDonald.*

## DISCUSSÃO

O presente estudo abordou a validação de conteúdos, aparência e semântica do protótipo “VivaCor PósOp”, apresentando resultados positivos que corroboram para sua aplicação como intervenção educativa em saúde.

Apesar do vasto potencial a ser explorado na esfera da *mHealth,* desafios técnicos podem afetar a aceitabilidade e viabilidade dos sistemas de telessaúde, como a necessidade de acesso à internet de boa qualidade^(34)^. No contexto brasileiro, o acesso à internet tem crescido nos últimos anos, alcançando cerca de 59 milhões de domicílios (82%) em 2021. No entanto, é importante ressaltar que a distribuição do acesso à internet fixa no país não é uniforme, sendo predominante nas regiões Sul e Sudeste e entre famílias das classes A e B^(35)^.

Assim, é essencial que as intervenções de telessaúde sejam adaptadas às necessidades e características do público-alvo, considerando também as diferentes realidades existentes no país, especialmente no contexto brasileiro, que apresenta uma ampla diversidade de realidades socioeconómicas e geográficas.

Para a presente validação, uma equipe multiprofissional foi convidada, com participação majoritária de enfermeiros, seguida de representantes de outras áreas. No entanto, não houve aceitação de colaboração por parte de profissionais médicos. Todavia, é importante destacar que outros estudos que abordaram a validação de aplicativos em saúde apresentaram resultados promissores, mesmo não contando com a avaliação de profissionais médicos^(36, 37)^, o que reforça a potência dos saberes multiprofissionais no cuidado em saúde. Ademais, a validação dos conteúdos por especialistas pode ser considerada um recurso fundamental para apoiar a concepção de intervenções de telessaúde^(38)^. Dessa forma, diante do cuidado multidisciplinar dos pacientes no pós-operatório de cirurgia cardíaca, sugere-se que estudos contínuos envolvam outras expertises, como médicos e também especialistas da área de tecnologia. Essa abordagem permite uma compreensão abrangente das necessidades e possibilidades de desenvolvimento de produtos tecnológicos para a saúde.

Em linha com a metodologia adotada para a validação do “VivaCor PósOp”, o estudo conduzido por Fernández-Gutiérrez et al.^(38)^ também se concentrou na validação do aplicativo mICardi, destinado ao autocuidado de pacientes com insuficiência cardíaca, visando aprimorar a qualidade de vida (QV). Nessa validação, houve o cálculo do IVC para cada tela do aplicativo, resultando em um IVC total de 0,851, indicativo de sua eficácia em melhorar a QV dos pacientes e promover a literacia em saúde. Assim como no estudo citado, o protótipo “VivaCor PósOp” foi desenvolvido com rigor metodológico, contando com a participação de especialistas no tema, o que fortalece a sua assertividade, sendo fundamental que esses recursos sejam construídos e validados utilizando metodologias robustas, a fim de garantir sua confiabilidade e transparência^(39)^.

Ainda que existam desafios técnicos, a telerreabilitação foi considerada pelos usuários um modelo alternativo e promissor à RC baseada em centros de saúde, conforme mostrado em uma revisão sistemática realizada por Subedi et al., na qual se observou alta aceitação da telerreabilitação por parte dos pacientes, devido à sua flexibilidade e facilidade de acesso. Além disso, intervenções que ofereciam sessões de automonitoramento e feedback individualizado foram avaliadas de forma positiva pelos pacientes^(34)^. Por isso, são necessários estudos futuros com o uso do “VivaCor PósOp” pelos pacientes submetidos à cirurgia cardíaca para avaliação de aceitação, automonitoramento e acompanhamento de saúde.

Ainda nesse cenário, a baixa motivação dos pacientes é uma questão comum na RC. A teoria da autodeterminação destaca a importância da satisfação das necessidades psicológicas relacionadas ao exercício, que está associada a uma maior motivação autodeterminada e comportamento de exercício mais eficaz^(40)^. Concomitantemente, a promoção do letramento em saúde representa uma oportunidade significativa para transformar a educação em saúde, exigindo uma abordagem proativa por parte dos pacientes^(41)^. Nesse sentido, é importante destacar a colaboração entre profissionais da saúde e tecnologia, bem como a participação ativa dos pacientes no desenvolvimento de recursos de *eHealth* ^(42)^. Essa abordagem centrada no usuârio permite compreender melhor as necessidades dos pacientes, como ocorreu na segunda fase de validação do protótipo do “VivaCor PósOp”, que coletou opiniões dos futuros usuários por meio de um questionário estruturado.

A validação semântica do protótipo “VivaCor PósOp” envolveu a participação de 10 pacientes. Na análise inicial, foi avaliada a consistência interna dos cinco domínios do instrumento de validação semântica, utilizando o coeficiente ômega de McDonald^(25)^. Os domínios objetivo, organização, linguagem e aparência apresentaram valores satisfatórios de ômega, todos acima de 0,70, conforme indicado por Campo-Arias et al.^(43)^. Entretanto, em relação ao domínio motivação, foi observado um coeficiente ômega de McDonald de 0,687, indicando uma correlação relativamente baixa entre os itens desse domínio. Além disso, os pacientes atribuíram a menor pontuação a esse domínio, com um escore de 0,933 para o IVC. Nesse sentido, é importante destacar que o construto da motivação é multifacetado, englobando a disposição do indivíduo em relação ao seu comportamento e suas consequências^(44)^.

Assim, um ponto relevante a ser considerado é o tempo decorrido desde as cirurgias dos pacientes da amostra, sendo que cerca de 50% deles haviam sido submetidos à intervenção cirúrgica há mais de cinco anos. A motivação é uma característica dinâmica, sensível a variações nas circunstâncias pessoais e sociais^(44)^, o que sugere que o período prolongado de pós-operatório pode ter influenciado os resultados observados neste domínio. Deste modo, é crucial implementar diversas estratégias para promover a motivação.

Um estudo de revisão sistemática conduzido por Chang et al. explorou intervenções de saúde baseadas no Modelo de Informação-Motivação-Habilidades Comportamentais (IMB), reconhecendo esses componentes como fatores determinantes para a adoção de comportamentos saudáveis e mudança comportamental. Após análise de 12 estudos, Chang et al. constatou que as estratégias mais comuns foram o uso de panfletos informativos, entrevistas motivacionais e vídeos educativos. Essas abordagens demonstraram resultados positivos, com uma melhoria na mudança de comportamento observada em 83,33% dos estudos analisados^(44)^.

Nesse sentido, a combinação de monitoramento remoto e acompanhamento individual tem demonstrado resultados positivos na redução da mortalidade e hospitalização de pacientes, especialmente em períodos de curto prazo^(45)^. Além disso, estratégias tecnológicas robustas aliadas a abordagens multidisciplinares podem contribuir para a promoção da mudança comportamental e melhorar os resultados de saúde. Assim, recomenda-se incluir modelos com aconselhamento personalizado, estabelecimento de metas específicas, medidas para promover o bem-estar psicossocial e orientações completas e atualizadas^(39, 46)^, sendo de fundamental importância o uso de métodos que garantam a confiabilidade e transparência dos dados nos aplicativos móveis^(39)^.

Por fim, na jornada do paciente, a transição do cuidado do ambiente hospitalar para o domiciliar desempenha um papel crucial na recuperação do indivíduo após a cirurgia cardíaca. Nesse contexto, a implementação de uma ferramenta de *mHealth* especialmente projetada para atender às necessidades dessa população emerge como uma solução inovadora para auxiliar este processo. É nessa perspectiva que o protótipo do aplicativo “VivaCor PósOp” foi desenvolvido e apresentou evidências de validade de conteúdo, aparência e semântica.

### Limitações do estudo

As limitações do estudo incluem a distribuição desigual de expertise entre profissionais de saúde, com predominância de enfermeiros, um número reduzido de pacientes no pré-teste e a avaliação das telas sem interação direta ou testes de usabilidade. A próxima fase do estudo se concentrará na programação do aplicativo, abordando detalhes relacionados aos sistemas, navegadores e adaptação de telas aos recursos utilizados, o que possibilitará investigações futuras para obter dados de usabilidade, bem como a validação clínica do aplicativo “VivaCor PósOp” com pacientes.

### Contribuições para a Área

O desenvolvimento de recursos *mHealth* adaptados às necessidades e características específicas do público-alvo, considerando as diversas realidades socioeconômicas e geográficas presentes no contexto brasileiro, emerge como uma estratégia fundamental. Nesse sentido, esses recursos têm o potencial de facilitar a transição dos cuidados hospitalares para domiciliares, proporcionando suporte tanto ao paciente quanto à família por meio de informações validadas por especialistas no tema, promovendo uma experiência mais personalizada e eficaz no contexto da saúde.

## CONCLUSÕES

O estudo focou no desenvolvimento do protótipo do aplicativo “VivaCor PósOp”, ressaltando sua capacidade de promover o autocuidado em pacientes após cirurgia cardíaca. Abordando elementos cruciais do pós-operatório e respaldado por evidências científicas, o aplicativo obteve validações satisfatórias tanto em conteúdo (RVC=0,86) quanto em aparência (IVA=0,99). A validação semântica, realizada com pacientes pós-cirurgia cardíaca, reforçou sua confiabilidade, apresentando um IVC total de 0,978. Estes resultados sublinham a importância do uso de tecnologias educacionais validadas, como o “VivaCor PósOp”, no cenário de saúde, destacando sua relevância como ferramenta promissora para favorecer o autocuidado e garantir um acompanhamento eficaz durante a recuperação pós-operatória.

## Data Availability

https://doi.org/10.48331/scielodata.ILIRZR
